# Recent Advances in Polysaccharide-Based Nanocomposite Films for Fruit Preservation: Construction, Applications, and Challenges

**DOI:** 10.3390/foods14061012

**Published:** 2025-03-17

**Authors:** Xin Chen, Xin Ding, Yanyan Huang, Yiming Zhao, Ge Chen, Xiaomin Xu, Donghui Xu, Bining Jiao, Xijuan Zhao, Guangyang Liu

**Affiliations:** 1 State Key Laboratory of Vegetable Biobreeding, Institute of Vegetables and Flowers, Chinese Academy of Agricultural Sciences, Key Laboratory of Vegetables Quality and Safety Control, Ministry of Agriculture and Rural Affairs of China, Beijing 100081, China; 2 National Center of Technology Innovation for Comprehensive Utilization of Saline-Alkali Land, Dongying 257347, China; 3 Key Laboratory of Quality and Safety Control of Citrus Fruits, Ministry of Agriculture and Rural Affairs, Southwest University, Chongqing 400712, Chinaxijuanzh@swu.edu.cn (X.Z.)

**Keywords:** food packaging, polysaccharide-based nanocomposite films, design and construction, fruit preservation

## Abstract

With the constantly escalating demand for safe food packaging, the utilization of biodegradable polysaccharide-based nanocomposite films is being explored as an alternative to traditional petrochemical polymer films (polyvinyl alcohol, polybutylene succinate, etc.). Polysaccharide-based films have excellent mechanical properties, water vapor transmission rates, and other physical characteristics. Films can fulfill numerous demands for fruit packaging in daily life. Additionally, they can be loaded with various types of non-toxic and non-biocidal materials such as bioactive substances and metal nanomaterials. These materials enhance bacterial inhibition and reduce oxidation in fruits while maintaining fundamental packaging functionality. The article discusses the design and preparation strategies of polysaccharide-based nanocomposite films and their application in fruit preservation. The types of films, the addition of materials, and their mechanisms of action are further discussed. In addition, this research is crucial for fruit preservation efforts and for the preparation of polysaccharide-based films in both scientific research and industrial applications.

## 1. Introduction

Fruits are abundant in multivitamins, which have antioxidant and free radical scavenging abilities. These are significant in anti-aging effects and are useful in the treatment of some chronic diseases [1]. Moreover, fruits have dietary fiber, which helps with intestinal motility, digestion of food, and balancing blood sugar. Fruits are also rich in carbohydrates and proteins. The nourishment makes them an ideal environment for bacteria and fungi. Additionally, the acidic condition of fruit is particularly suitable for the growth of yeasts and molds. However, the factors inducing fruit spoilage include not only various types of pathogenic bacteria but also mechanical damage to the fruit. The interaction of various enzymes (polyphenol oxidase, pectinase, etc.) within the fruit itself is also a key factor in fruit spoilage. To address the issue of fruit spoilage, people usually use freshness preservation methods to extend shelf life. The common methods of fruit preservation include chemical preservation and physical preservation. Chemical preservation commonly involves treating fresh produce with chemical compounds such as electrolyzed oxidizing water. Some of these compounds are potentially harmful to humans, including hydrogen sulfide, ozone, ethanol, etc. [2]. In terms of physical preservation, in order to preserve the quality of fruits, multiple packaging methods have been deployed to address preservation requirements, such as active packaging, active coating, and modified atmosphere packaging (MAP). Polysaccharide-based nanocomposite films, created by incorporating various nanocomposites and bioactive compounds using polysaccharides as a substrate, show promising application prospects for research on fruit preservation [3,4]. Additionally, biopolymers have shown strong application prospect in several areas, such as medicine [5], environment [6], energy [7], and construction [8]. Consequently, the advancement of biopolymers enhances food preservation capabilities and plays a pivotal role in driving industrial modernization and fostering sustainable development across various sectors.

**Figure 1:**
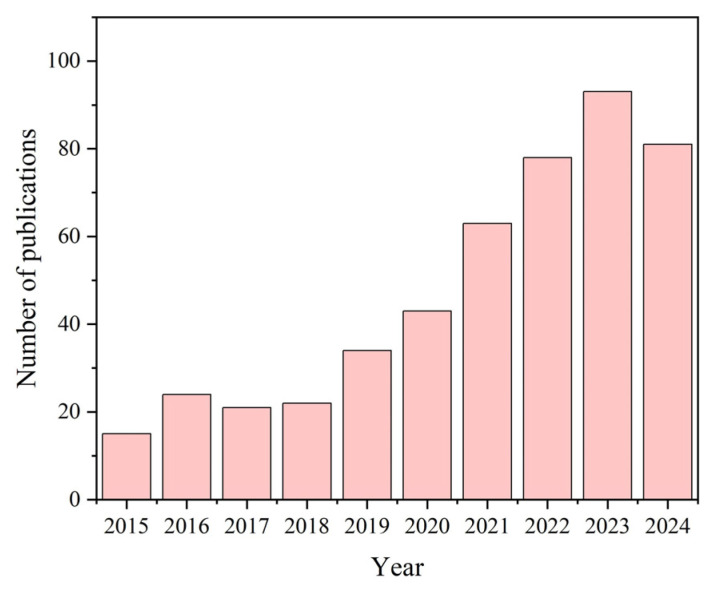
Trends of research article publications in the field of polysaccharide films and fruit packaging in the last decade (data from the Web of Science with themes of “polysaccharide films” and “fruit packaging”).

Food packaging is vital for food safety, storage, and freshness. Conventional packaging produces microplastics that have an impact on the environment. Developing biopolymer films (from polysaccharides) for food packaging applications contributes to the development of sustainable packaging materials, thereby reducing the dependence on non-biodegradable plastics and mitigating environmental pollution. Polysaccharide films demonstrate moderate preservative efficacy, primarily through moisture retention, and gas barrier properties [9]. However, during storage or transportation, fruits are susceptible to water loss, infestation by pathogenic bacteria, and oxidation. Their physical characteristics such as mechanical properties, water vapor transmission rate, and so on are insufficient meet the application of freshness preservation for fruits. Key physicochemical properties such as mechanical strength, surface hydrophobicity, and the water vapor transmission rate of the films can be considered to be improved by adding metal–organic frameworks (MOFs) or bioactive substances to the film. In order to improve some of chemical and biological characteristics of polysaccharide-based films, many researchers have begun to refer to green, renewable composites, bio–metal–organic frameworks (Bio-MOFs) [10]. Green, renewable composites materials have garnered significant interest as a novel category of porous materials [11]. Polysaccharide-based nanocomposite films are positioned as promising candidates for food packaging applications, positioning them as promising candidates for sustainable and functional packaging solutions in the food industry. These intelligent packaging systems integrate natural polysaccharides with nanomaterials to establish a multifunctional protective microenvironment. They possess inherent antimicrobial properties through controlled release of bioactive components [12] and senescence delay via modified atmosphere regulation [13]. Even smart packaging can monitor the commodity value of fruit in real time through the color of film that changes as the environment changes [14]. Future research may focus on the dynamic evolution of flavor profiles, textural properties, and nutritional composition within advanced encapsulation matrices, alongside thermoresponsive pest control mechanisms during seasonal temperature fluctuations [15]. Recently, the application of polysaccharide-based nanocomposite films has become a research hotspot (Figure 1).

## 2. Factors and Mechanisms Affecting Quality Deterioration of Water Fruits and Preservation Methods

### 2.1. Fruit Ripening

Fruit quality deteriorates rapidly and dramatically over time, impacting postharvest shelf life and consumer acceptance [16]. Fruit ripening is a genetically programmed and environmentally regulated process involving complex biochemical and physiological changes. For instance, climacteric fruits like apples exhibit a peak in endogenous ethylene production during ripening, which is tightly controlled by genetic factors [17].

NAC (NAM, ATAF1/2, and CUC2) transcription factors (TFs) have been found to facilitate various developmental processes in plants. For example, ripening-inducing factor (FaRIF) has been identified and demonstrated through multiple experiments in which FaRIF controls key processes of strawberry ripening such as fruit ripening, pigmentation, and accumulating sugar. FaRIF plays a crucial role in regulating strawberry ripening from the initial growth phases, overseeing the biosynthesis and signaling of abscisic acid, cell wall degradation, and modification, the phenylpropane pathway, production of volatile compounds, and maintaining the balance between aerobic and anaerobic metabolism. Thus, fruit ripening is dominated by a combination of multiple factors [18].

### 2.2. Fruit Deterioration

After ripening, under temperature stress, oxygen stress, biotic stress, and other stresses, there will be a deterioration of the commercial properties of the fruit, such as loss of fruit flavor, changes in fruit texture, as well as rotting or even the production of off-flavors. These changes are not solely due to endogenous biochemical processes but are accelerated by chemical interactions with environmental factors, such as ethanol (fermentation metabolism), hexanol, octanol, heptanol (lipid oxidation), methionine, methanethiol, dimethyl disulfide (oxidative degradation of sulfur-containing amino acids), and other metabolites, which result in the commercial deterioration of the fruit [19].

It is worth noting that the fruit surface has an outer layer that is hydrophobic and insulated from microorganisms and oxygen, and intact fruit skin acts as a hydrophobic barrier against microorganisms and oxygen, delaying spoilage. However, mechanical damage compromises this protection, increasing susceptibility to decay. According to previous studies, microbial load and decay in strawberries increased after mechanical damage to the fruit. Not only that, when the environmental temperature increased by 1 °C, the collision damage volume and damage volume percentage of the strawberries increased by 6.4658 mm^3^ and 0.0348% [20].

### 2.3. Methods of Fruit Preservation

Scientists have explored a variety of preservation methods with the purpose of solving the above problems of spoilage and deterioration. First of all, MAP extends freshness by altering the gas composition surrounding the fruits. The fruits wrapped in packaging benefit from modified atmosphere packaging, which is highly effective in preventing microbial growth and temperature changes within the package. It can also isolate oxygen to hinder the oxidation of the fruit, and modified atmosphere has been widely popularized in the field of fruits [21], cold meat, and cooked food [22]. The disadvantages of modified atmosphere packaging are also apparent. Poor modified atmosphere packaging can lead to more rapid spoilage of the product, and inappropriate CO_2_ concentrations may cause drip loss, accelerating deterioration, which is a continuous process that will accelerate the deterioration of the fruit. However, the price of modified atmosphere packaging equipment is too expensive for some producers, and plastic waste is generated by modified atmosphere packaging. It will have an impact on the environment.

In addition, food irradiation is a non-thermal technique that involves exposing food in a specific way. Food irradiation utilizes either non-ionizing radiation (e.g., UV, visible light) or ionizing radiation (e.g., gamma rays, X-rays) to inactivate microbes. This process effectively destroys microbes, such as viruses and bacteria, in food and agricultural commodities [23]. UV-B-treated samples increased the content of hydroxycinnamic acid but decreased flavonol and induced the production of antioxidants in the fruit [24]. Among the thermal technologies, pulsed light (PL) has emerged as an innovative sterilization approach for fruits and fruit juices. PL can inactivate the Browning polyphenol oxidase in fruits, and the inactivation rate can reach 0.0286 cm^2^/J at pH 4.0 [25]. Over the past decade, the antimicrobial effect of PL treatment on common bacteria, yeasts, molds, spores, and some pathogenic microorganisms on the surface of fruits and vegetables has been well documented [26,27,28,29,30]. Also, for a novel approach to fruit preservation, coatings with various bioactive compounds can improve antimicrobial and antioxidant properties compared to single-material coatings [31]. Due to the physical properties of the coatings, which are in the gel state, they are advantageous for preserving fruits such as lychee and longan, but for small berry fruits, completely removing the coating prior to consumption remains a challenge [32,33]. Heat treatment involves exposing the fruit to higher temperature water or air for a period of time. It can be an effective solution for disease management and rot prevention and browning. But the parameters, including exposure time and temperature, must be carefully monitored, as prolonged exposure can negatively impact fruit quality [34].

Polysaccharide-based nanocomposite films are a cost-effective and eco-friendly preservation method for fruits, vegetables, and meats. Their performance surpasses conventional packaging, aligning with China’s ecological policies [35,36]. By combining various polysaccharides to create a composite film and incorporating biologically active substances with antimicrobial and antioxidant effects, we can address the shortcomings of single films such as curcumin, tea polyphenols, etc., and nanomaterials that can be loaded with bioactive substances. Biological substances not only can greatly improve the film’s antimicrobial effect of freshness preservation but also can be utilized to observe the freshness of foods using changes in pH or temperature [37]. The packaging of polysaccharide-based nanocomposite films has great potential in the market for consumers and businesses [38,39].

#### Fundamental Mechanisms of Polysaccharide-Based Packaging

The preservation efficacy of polysaccharide-based nanocomposite films stems from their multifunctional barrier properties and bioactive interactions, as evidenced by recent advances in food packaging nanotechnology [40]. The gas barrier mechanism primarily arises from the tortuous path architecture created by nanofillers (e.g., cellulose nanocrystals, montmorillonite) within the polysaccharide matrix [41]. The integration of young apple nanofibers as dual-functional electrostatic crosslinkers and toughening agents significantly enhanced the hydrophobicity and mechanical properties of chitosan films. This modification caused the improvement of water contact angle, elongation at break, and tensile strength. Furthermore, the barrier performance of the film was significantly upgraded, with notable reductions in water vapor permeability and oxygen permeability, as well as enhanced UV-A blocking capability [42]. In addition to the physical properties of the film, which are enhanced by the blending of different polysaccharides, the addition of bioactive substances has also been shown to greatly improve the film’s properties. It has been reported that the process of plasticization is characterized by the formation of new hydrogen bonds between the plasticizer and polymer molecules. This is achieved by the distribution of the plasticizer within the gaps of the polymer network structure, thereby disrupting the original hydrogen bonds between polymer molecule chains. At present, four main theories are employed to explain the plasticizing mechanism of plasticizers: lubricity theory, gel theory, free volume theory, and mechanical theory [43,44]. Incorporation of citric acid (CA) as an esterification agent into hemicellulose resulted in the formation of crosslinked structures through esterification reactions. In comparison with the 41.6° water contact angle of unmodified films, that of CA-modified hemicellulose films increased to 87.5° [45]. Antioxidant functionality emerges from both inherent polysaccharide properties and nano-encapsulated active compounds. Chitosan/starch fibrous films functionalized with clathrate compounds exhibited enhanced antibacterial activity in vitro and on cherry surfaces, with a sustained release of potassium cinnamate for 240 h. The potential benefits of extended sustained release include the control of the duration of film freshness of the fruit, as well as the avoidance of direct contact of large quantities of bioactives with the fruit, and the prolongation of freshness. The incorporation of clathrate compounds significantly improved the film’s thermal stability and hydrophilicity while reducing its crystallinity and brittleness [46].

## 3. Polysaccharide-Based Nanocomposite Film Design and Preparation Strategy

**Figure 2:**
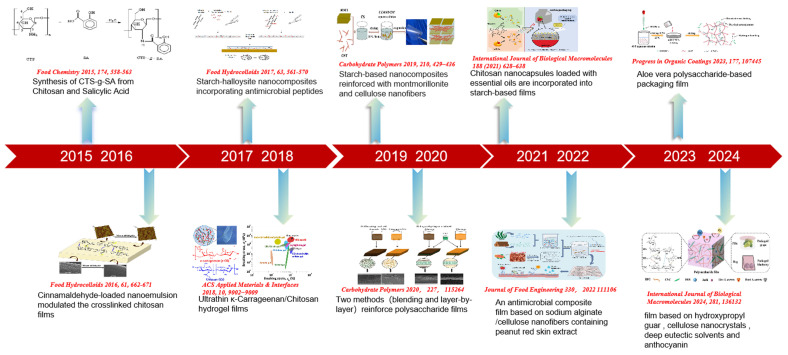
The timeline of polysaccharide-based nanocomposite films in the field of surgery [52,53,54,55,56,57,58,59,60,61].

After learning about the various ways of preserving fruits, it was felt that the application of polysaccharide-based nanocomposite films shows promising prospects. In recent years, polysaccharide-based nanocomposite films have been widely surveyed in a variety of fields (Figure 2). The formation of films involves electrolysis, ionic cross-linking, or the formation of hydrogen bonds through the decomposition of biopolymers [47,48,49]. For the purpose of achieving excellent performance for polysaccharide-based films, it is necessary to understand the basic properties of various polysaccharides, as well as the series of improvements and applications that occur in films after incorporating bioactive compounds or nanomaterials [50,51]. Therefore, the article will synthesize various aspects such as types of polysaccharide-based nanocomposite films, loaded bioactive compounds, and so on (in Table 1).

### 3.1. Polysaccharide Types

#### 3.1.1. Starch

**Figure 3:**
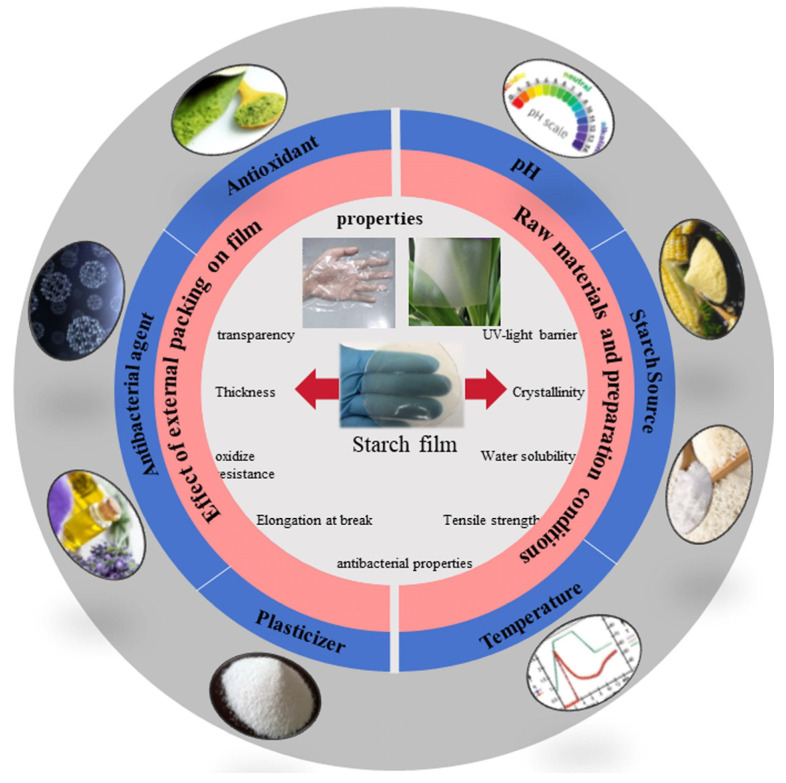
Factors affecting the properties of starch-based films [71].

Starch includes potato starch, sweet potato starch, lily starch, and many other polysaccharides derived from plants. Starch is considered a highly favorable natural polymer due to its intrinsic biodegradability, abundant availability, and yearly renewability. Utilizing starch-based polymers offers an appealing and cost-effective foundation for innovative polysaccharide-based films, given their low material expenses and compatibility with standard plastic-processing machinery [71] (Figure 3).

Potato, for example, is the world’s fourth largest food crop and a common vegetable in the human diet. Potato starch, sourced from potato tubers, exhibits superior pasting temperature, viscosity, transparency, and other physicochemical characteristics compared to other starch varieties [72]. The content of straight-chain starch in potato starch is only 20–33%, with the remainder being branched-chain starch. The proportion of straight-chain starch significantly influences key film properties such as tensile strength, film-forming ability, and transparency [73,74]. It has excellent thickening, sustained release, adhesion, and hydrophilic properties. However, simple potato starch films typically exhibit poor water vapor barrier properties due to their high water retention capacity. While starch-based films have high oxygen barrier properties, they often fail to meet the requirements for freshness and antimicrobial functionality. The hydrophilicity, tensile strength, and other physical properties of potato starch films can be altered by the addition of certain materials [75,76]. For example, doping different concentrations of potato peel polyphenol (PP) or chitosan nanoparticle–potato peel polyphenol (CNP-PP) into starch films resulted in an increase in their opacity and enhanced hydrophobicity. X-ray diffraction (XRD) and scanning electron microscopy (SEM) indicated that after incorporating PP, the composite film’s surface exhibited a smooth and tightly packed structure, with a flat and dense cross-section. The addition of CNP-PP resulted in a significant increase in the elongation at break of the films (*p* < 0.05), accompanied by a decrease in tensile strength. This was mainly due to the reduction in crystallinity of CNP-PP and the distribution of agglomerates on the film surface [77]. In addition to this, the addition of PP to potato starch was utilized to increase the ductility of the film by interweaving the macromolecular chains through hydrogen bonding and electrostatic forces using the casting method.

The casting method was employed to successfully fabricate corn starch films, and a series of tests were conducted to investigate the effects of varying the cinnamaldehyde ratio on the material properties of corn starch films. The findings demonstrated that the water vapor transmittance rate of the films decreased as the cinnamaldehyde ratio increased [9]. Incorporation of citrus-derived cellulose fibers into starch-based composite films resulted in a significant reduction of water vapor permeability (WVP) to 3.46 × 10^−10^ g·m^−1^·s^−1^·Pa^−1^, coupled with remarkable enhancement of UV-barrier properties demonstrating 99.29% UV-blocking efficiency [78]. It is worth noting that fruits such as citrus fruits require higher levels of water to ensure their commercial value, but fruits such as berries should avoid water loss as much as possible. Therefore, the water vapor transmission rate can be controlled by adding different amounts of bioactive substances to achieve the desired effect on different fruits [79].

#### 3.1.2. Cellulose

**Figure 4:**
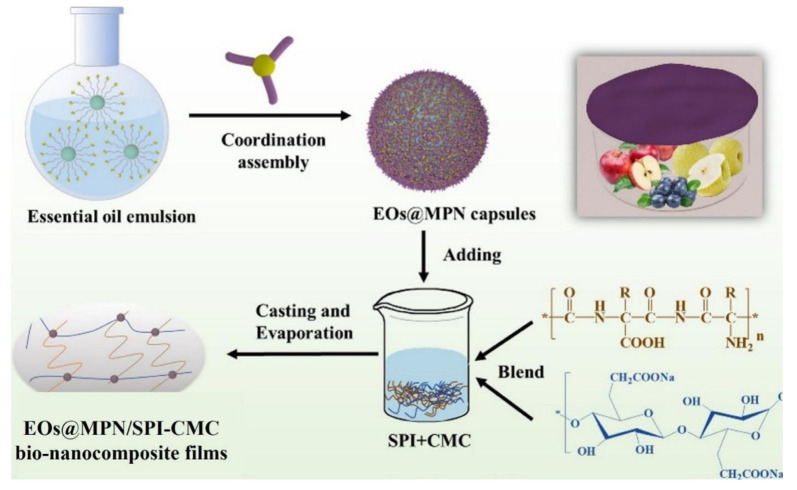
Biocompatible Fe^III^–tannic acid-based metal phenolic networks (MPNs) encapsulate natural essential oils (EOs) in EOs@MPN nanocapsules. These were incorporated into blend films of soy protein isolate (SPI) and CMC to create multifunctional bio-nanocomposite films for food preservation [81].

Cellulose, a linear polysaccharide made up of glucose residues connected by β-1,4 glycosidic bonds, is a key component of cell walls in plants of all types. It is the most abundant renewable carbon source, with excellent film-casting properties, biodegradability and non-toxicity. Its distinctive physical and chemical characteristics, such as oxygen barrier capability, film-forming ability, and elevated water retention capacity, render it an excellent material for active food packaging applications. Consequently, cellulose has found widespread application in the food and pharmaceutical (Figure 4), agriculture, and wastewater treatment sectors. Many bioactive substances have been added to the films for improving the physicochemical properties of cellulose-based films. There have been previous attempts to crosslink carboxymethyl cellulose using 10% citric acid (CA) to improve the water solubility of carboxymethyl cellulose (CMC) [80]. The thermal stability of protein/polysaccharide composite films is improved by mixing gelatin with carboxymethyl cellulose; it also improves the stability of the film when various substances are added to form an effective combination with it [80,81,82,83,84] (Figure 4).

Additionally, we classify cellulose into regenerated cellulose, wood pulp, microcrystalline cellulose, carboxymethyl cellulose, and hydroxyalkyl cellulose. The fabrication of polysaccharide-based nanocomposite films can be tailored for specific purposes by leveraging the unique characteristics of different types of cellulose. For example, hydrophilic and hydrophobic cellulose materials have been used to fabricate hydrophilic and hydrophobic films, respectively [85]. A composite polysaccharide film synthesized using lignocellulosic nanofibers (LCNFs) and wheat gluten (WG) showed outstanding freshness preservation performance for six different respiratory metabolisms in the aspect of prolonging the shelf-life of the fruits, displaying excellent oxygen barrier properties (15.9 cm^3^ μm^−2^ day^−1^ kPa^−1^) and water vapor evaporation (10.3 g mm^−1^ m^−2^ day^−1^) [86].

CMC derivatives demonstrate versatile modifiability through diverse approaches to enhance functional characteristics [87]. A notable advancement involves the TEMPO (2,2,6,6-tetramethylpiperidine-1-oxyl radical)-mediated oxidation of cellulose nanofibril carboxyl groups, which facilitates the conversion to carboxylated nanofibrillated cellulose (CNF) [88]. Experimental evidence reveals that incremental incorporation of carboxymethylated cellulose (0–12 wt%) in starch-based composite films significantly improves mechanical performance, with tensile strength escalating from 23.89 MPa to 38.37 MPa and elongation at break increasing from 21.00% to 27.31% [67]. Subsequent investigations employing cold plasma modification—a physical surface treatment—demonstrate enhanced antimicrobial and antioxidant functionalities in CMC-based films, potentially mediated through alterations in water vapor permeability (WVP) and surface topography that modulate fruit respiration and transpiration processes [89]. Beyond these modifications, advanced chemical derivatization strategies, including acetylation [90], silylation [91], and ethylation [92], have been systematically developed to tailor cellulose films for specific preservation requirements. Particularly, acetylation modification effectively enhances CNF solubilization through disruption of intermolecular hydrogen bonding networks, thereby promoting the fabrication of rapidly biodegradable matrices. These multi-scale modification approaches, spanning from molecular-level chemical functionalization to macroscopic surface engineering, establish a robust foundation for developing sustainable and performance-tunable food packaging materials that address diverse preservation challenges while maintaining environmental compatibility [93].

#### 3.1.3. Chitosan

**Figure 5:**
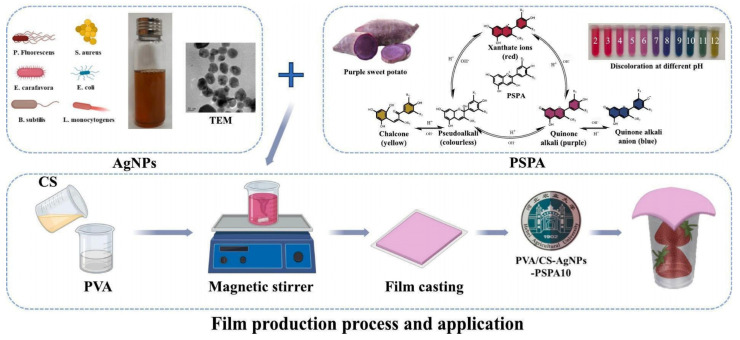
Synthesis and design of chitosan-based smart packaging [104].

Chitosan (CS) is recognized for its excellent film-forming, degradable, and bacteriostatic properties. In addition, chitosan films exhibit effective barriers against the permeation of water vapor and oxygen. Reducing oxygen permeation helps decrease the respiration rate in fruits and vegetables, thereby extending the shelf life of food products [94]. However, the relatively lower mechanical strengths and barrier properties of chitosan films restrict their potential for use in freshness preservation applications. Therefore, the structural and functional properties of CS films must be enhanced to meet the requirements for multifunctional active packaging materials [95]. Researchers have already enhanced CS films by adding bioactive substances such as tea polyphenols (TP), zeaxanthin, curcumin, and cinnamon essential oil, as well as various nanomaterial modifications such as γ-cyclodextrin–metal–organic frameworks (γ-CD-MOFs), nanohybridized particles of TiO_2_ [96], Zn nanoparticles, etc. The incorporation of inorganic nanoparticles with bioactive substances in CS results in higher levels of barrier, mechanical, thermal, and antimicrobial characteristics for composite packages [97,98,99,100]. Thus, polysaccharide-based nanocomposite films combined with bioactive compounds and MOF materials have been widely used in fruit preservation applications, and in addition to the use of single chitosan-based films, polysaccharide-based films in combination with other polymers have been equally widely used to maintain postharvest quality, minimize spoilage, and prolong the shelf-life of a wide range of fruits and vegetables [101]. Adding polyphenolic to CS promotes the increase of interfacial energy between hydrophobic fruit surfaces rich in phenolic hydroxyl groups and the film, for instance, the coupling of the polyphenol gallic acid (GA) to CS. Cherry tomatoes were preserved using the films, and after 8 days, the hardness of the coated samples was 16.9 ± 0.36 N and the hardness of the uncoated samples was 5.87 ± 0.02 N. Compared to uncoated samples, film-coated cherry tomatoes exhibited a lower rate of mass loss and higher hardness. The results indicated that CS-GA effectively preserved the freshness of fruits and made them suitable for food packaging [102]. Multifunctional edible composite films based on branched starch/chitosan were prepared by synthesizing zinc oxide nanoparticles (ZnONPs) and propolis. The composite films exhibited strong antimicrobial activity against *E. coli* and *Listeria monocytogenes* and showed excellent antioxidant activity [103]. As shown in Figure 5, the incorporation of purple sweet potato anthocyanin (PSPA) and silver nanoparticles (AgNPs) into chitosan not only effectively provides antimicrobial and mechanical properties but also allows the use of the color change in PSPA to determine whether the fruit is edible [104].

The rational design of nanocomposite systems integrating metallic nanoparticles with bioactive phytochemicals has been advanced as a means of functionalizing food packaging materials [105]. Chitosan-SeNP composite films enhanced thermal stability (21% TGA mass loss), radical scavenging capacity (58.75% DPPH inhibition), and biosafety (100% cell viability), effectively suppressing microbial proliferation (>60% fungal reduction) in tomato preservation while maintaining chromatic stability [106]. A particularly noteworthy experiment involved synergistic integration of the photocatalytic antimicrobial property of nano-TiO_2_ with the antioxidant property of chrysanthemum essential oil, resulting in a significant enhancement of the composite film’s antibacterial and antioxidant properties, as well as its mechanical strength. In the context of preserving *Actinidia arguta*, the composite film effectively regulated respiration rate, inhibited microbial growth, and preserved nutrients such as vitamin C and titratable acid, thereby extending the shelf life to 10 days [107]. Furthermore, the study systematically evaluated the difference in the freshness preservation effect of films under light and dark conditions, providing a scientific basis for real-world storage conditions. Beyond single-component modifications, advanced structural designs such as vanillin/tetramethylphosphonium chloride dual-crosslinked networks yielded exceptional mechanical performance (95.89 MPa tensile strength). Mechanistic studies revealed synergistic interactions between quaternized lignin’s ammonium groups and ZnO nanoparticles, enhancing both antimicrobial efficacy and antioxidant capacity. The present study has demonstrated that the application of nanocomposites has the potential to extend the shelf-life of *Actinidia arguta* (10 days) and grapes (12 days) through the modulation of respiratory rate and the preservation of nutrients [108]. However, polysaccharide-based nanocomposite films still present serious challenges in terms of adaptability to different environments, migration of nanomaterials, and large-scale production. The current research paradigm remains constrained by insufficient validation across diverse fruit matrices.

#### 3.1.4. Seaweed Polysaccharides

Seaweed polysaccharides are used in industry as a form of hydrocolloids. Their derivatives, such as alginate [109], carrageenan [110], and agar [111], are employed in the food industry as thickeners, gelling agents, and food stabilizers [112]. Alginate, a naturally occurring anionic polysaccharide derived from brown seaweed, exhibits relative stability within the pH range of 4–10. Beyond its application in composite manufacturing, alginate finds extensive use in biomedical and electrical fields owing to its low cost, high biodegradability, and minimal toxicity [113,114]. Carrageenan is typically extracted from a specific class of red seaweed. It is a safe and natural polysaccharide that has been approved for use as a food additive. The applications of carrageenan in the food and pharmaceutical industries have been extensively documented, including its roles as a viscosity modifier, stabilizing agent, and emulsifier. Carrageenans are categorized into three types (λ, ί, and κ) based on the varying contents of 3,6-anhydro-α-D-galactopyranosyl units and sulfate esters [115]. Agar was the first alginate used in human food formulation [116]. The chemical composition of agar primarily comprises two heteropolysaccharide components: agarose (the gelling component) and agaropectin (the non-gelling component). Typically, the latter is removed during the production process, resulting in agar powder, which serves as the raw material for polysaccharide-based nanocomposite films [117,118]. One major issue with alginate polysaccharide biofilms is which subpar performance in terms of mechanical strength and water vapor barrier properties when compared to traditional non-renewable polymers.

**Figure 6:**
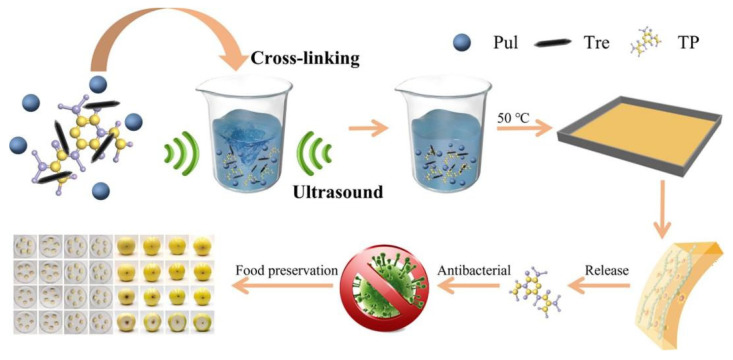
Incorporating tea polyphenol-loaded pullulan/trehalose (TP@Pul/Tre) into a composite film for fruit preservation [121].

Hence, seaweed polysaccharides are usually blended with other components to improve the quality of seaweed films, including the incorporation of nanoparticles. Figure 6 illustrates the general production process of seaweed-based nanoparticle films. Sodium alginate (SA) is another polysaccharide rich in hydroxyl and carboxyl groups, showcasing outstanding adsorption capabilities for heavy metal ions and being well-suited for the production of absorbent materials. In the fields of food packaging, alginate-based films have the advantage of superior biocompatibility, biodegradability, water absorption, gelation, film formation, and non-toxicity. The use of SA-based food cling film has great practical value. It has been demonstrated by using SA-based food cling film with chitosan [119], sucrose [120], pullulan [121], starch, and other polysaccharides to increase the fluidity of polymer chains and improve the mechanical properties of natural polymer films [122]. However, in order to increase the antimicrobial properties of alginate-based films, natural antimicrobial agents and antimicrobial nanostructures have been used, where natural antimicrobial agents include EOs of oregano, cinnamon, or eucalyptus in order to prolong the shelf-life of packaged fruits. Antimicrobial nanostructures, including metallic and non-metallic nanomaterials, are nanomaterials that ensure the safety of packaged products. The incorporation of Laurel Leaf Extract (LLE) and Olive Leaf Extract (OLE) into alginate films reduces the moisture content and WVP value of the films, which may be related to the hydrophobicity of the extracts. In addition, the simultaneous incorporation of LLE 1% (*w*/*v*) and OLE 1% (*w*/*v*) greatly improved the inhibition of *S. aureus* [123]. Utilizing the layer-by-layer assembly method (LBL) to integrate the photoresponsive QDs@ZIF-8 nanocomposites (NPs) into CS/SA-based films, the addition of QDs@ZIF-8 enhanced the antimicrobial performance of CS/SA composite films, which was associated with the generation of reactive oxygen radicals (ROS) by QDs@ZIF-8 upon light irradiation. According to the experimental results, it is known that the antimicrobial performance of CS/SA/QDs@ZIF-8 film-treated kiwifruit had a significantly longer shelf life [69].

Sodium alginate (SA) films have been shown to exhibit superior transparency, structural homogeneity, and low oxygen permeability in comparison to their chitosan-based counterparts [106]. However, their practical utility is constrained by their inherent hydrophilicity and excessive water vapor transmission rates (2132.57 g·m^−2^·d^−1^). Nanocomposite engineering strategies, such as SA/AgNPs integration, have been shown to effectively mitigate these limitations by reducing water vapor permeability to 1922.47 g·m^−2^·d^−1^ while enhancing tensile strength to 5.96 MPa. Recent innovations leverage electrostatic interactions between protonated basic amino acids (e.g., lysine) in tea seed cake protein (CCP) and SA’s carboxyl groups, generating dense networks that elevate tensile strength to 20.83 MPa (188% improvement over pristine SA) and reduce water vapor permeability by 32%. The aromatic residues (phenylalanine/tyrosine) in CCP have been shown to confer UV-blocking efficacy (<7% transmittance at 300 nm) and potent antimicrobial activity (83% and 91% inhibition against *E. coli* and *S. aureus*) [124]. Notably, incorporating pterostilbene nanoemulsions via high-pressure homogenization into chitosan/SA matrices achieves simultaneous enhancement of oxygen barrier (26% reduction to 50.88 cm^3^·m^−1^·24 h^−1^·0.1 MPa^−1^), amplification of radical scavenging (4-fold ABTS+ inhibition), and 99% MRSA suppression. However, critical barriers hinder commercialization, including the absence of defined nanoparticle migration–toxicity correlations (ZnO, Fe_2_TiO_5_), the energy-intensive processing (homogenisation/solvent casting), and batch variability in biopolymer feedstocks [125]. Advancements in continuous fabrication technologies, particularly electrospinning and 3D printing, have the potential to bridge the gap between functional prototyping and scalable, safety-certified production of alginate-based packaging systems.

### 3.2. Addition of Nanomaterials

Antimicrobial nanomaterials of metals and their oxides (titanium dioxide, zinc oxide, silver, copper oxide), non-metallic nanomaterials (silicon dioxide), and other nanomaterials (composite nanomaterials, nanocellulose) have usually been added to polysaccharide-based films to enhance the antimicrobial properties of the films [126]. The main antimicrobial mechanism of antimicrobial nanomaterials is that they can electrostatically bind to the microbial cell wall, altering the film potential, which leads to film damage, which in turn leads to impaired respiration, transport imbalance, disruption of energy transduction, and cell lysis, ultimately leading to cell death. Another mechanism of nanostructured antimicrobial agents is based on the generation of ROS from nanomaterials [127].

#### 3.2.1. Metal and Metal Oxide Nanomaterials

Zhang et al. [127] used walnut green hull polysaccharide as raw material and added silver nanoparticles (AgNPs). The experimental results showed that AgNPs can effectively suppress the growth of *Escherichia coli* and *Staphylococcus aureus* at a concentration of 50 μg/mL, and the antibacterial effect of the film was better with the increase in AgNP addition [128]. Tang et al. [61] used a soluble casting method to load ZnO nanoparticles into composite films doped with microfibrillated cellulose (MFC) and soluble soybean polysaccharide. The UV transmittance of the films doped with nano zinc oxide (nZnO) was considerably lower than that of the undoped films, and this change was attributed to the strong UV absorption and scattering properties of nZnO. Similarly, the nZnO-doped nanocomposite films showed significant bacterial inhibition in the antimicrobial activity inhibition zone experiments, while the other nZnO-undoped composite films did not show any inhibition zone [129]. TiO_2_ nanoparticles are also effective in enhancing the properties of the film. However, direct doping of single TiO_2_ nanoparticles into the film can lead to particle aggregation, which impacts the film by reducing its tensile strength and antimicrobial properties. Ding et al. [96] prepared a series of composite films using the carbon-based material chitosan–ferulic acid (CF) and chitosan–ferulic acid–titanium dioxide nanohybrid particles (CFT NPs) as fillers. Among these, the shelf life of bananas and strawberries packaged with CFT (0.40 mg/mL) films was extended [130].

##### Silver Nanoparticles (AgNPs)

AgNPs have received more interest in applications for fruit preservation. The incorporation of AgNPs into chitosan matrices demonstrated remarkable antifungal efficacy, achieving complete inhibition (100%) of chilli anthracnose pathogen Colletotrichum truncatum. This nanocomposite system effectively mitigated anthracnose infection in chili peppers (Colletotrichum truncatum), showcasing its dual functionality as both a postharvest preservation agent and a potential preharvest disease management strategy for perishable crops [131]. AgNPs exhibit broad-spectrum antimicrobial activity through multiple mechanisms, including film disruption and reactive oxygen species generation. The integration of Hylocereus undatus (dragon fruit) extract with AgNPs via phytochemical-mediated reduction enhances biodegradation kinetics (the blended film demonstrated outstanding antimicrobial properties against *S. aureus* and *E. coli* by eliminating more than 99.99% after 6 h) [132]. The use of plant extracts/AgNPs as an alternative to chemical preservatives will be a future trend in food preservation, but the future concern will be more about how to avoid AgNP migration into food.

#### 3.2.2. Non-Metallic Nanomaterials

Mesoporous SiO_2_ nanoparticles have been extensively researched for their large surface areas and large pore volumes, which can enhance the encapsulation of bioactive compounds and enable controlled release. For instance, when a biologically active compound is encapsulated into konjac glucomannan (KGM) with SiO_2_ nanoparticles, the tensile strength of the hybrid film significantly improved compared to the pure KGM film, indicating enhanced mechanical properties [133]. Additionally, the tensile strength of hybrid films exhibited a notable enhancement by incorporating magnetic fibers. Moreover, the antimicrobial and antioxidant properties of polysaccharide-based films can be enhanced by adding magnetic SiO_2_ nanomaterials. For example, chitosan films doped with biocomposites exhibit stronger antimicrobial effects against *Bacillus cereus* than chitosan films [134], which can enhance the film’s ability to preserve fruit freshness [135]. Furthermore, Carlos Velasco-Santos prepares graphene-reinforced nanocomposites of chitosan–starch and carboxymethylcellulose–starch using a casting/solvent evaporation method, demonstrating the potential of graphene materials in polysaccharide-based nanocomposite films.

##### Graphene Oxide

Graphene oxide (GO) serves as a structural stabilizer in polymeric films through its lamellar architecture. The interfacial interaction between GO nanosheets and polymer matrices generates hierarchically porous networks, enhancing water transport channels while improving surface hydrophilic properties. When integrated with metallic nanomaterials (Ag, ZnO), GO-based nanocomposites exhibit synergistic bacteriostatic effects through combined physical barrier and ion-release mechanisms [38,136]. Furthermore, GO’s oxygen-rich functional groups enable efficient encapsulation of volatile compounds like essential oils via hydrogen bonding interactions, achieving sustained release profiles dominated from the carrier matrix. This encapsulation system retained 73.7% release efficiency at 45 °C, demonstrating thermal-responsive delivery capabilities. These multifunctional attributes position GO as a promising candidate for developing intelligent packaging systems with environmental-adaptive properties [137].

##### Cellulose Nanocrystals

Cellulose nanofibers (CNFs) form structurally integrated nanocomposites with biopolymer matrices through multifunctional crosslinking mechanisms, including dynamic Schiff base formation, metal–ligand coordination, and hierarchical hydrogen-bond networks. The CNF–chitosan composite demonstrated synergistic functionalities: exceptional antioxidant capacity (93.6% DPPH radical scavenging) and UV blocking efficiency (>90%), coupled with optimized gas barrier properties (oxygen transmission rate: 0.9 cm^3^·μm/(m^2^·day·kPa; water vapor permeability: 456.94 g/(m^2^·24 h)) [138]. When complexed with pectin, the system exhibited enhanced mechanical strength (23.09 MPa tensile strength), hydrophobicity (water contact angle: 91.23°), and WVP (1.02 × 10^−12^ g/cm·s·Pa) [139].

#### 3.2.3. Controlled Release of Nanomaterials

In addition to the previously mentioned antimicrobial properties of nanomaterials and the enhanced mechanical properties of films, nanomaterials can also effectively encapsulate biologically active substances to achieve a slow-release effect. When the nano-network structure decomposes under specific pH [140], enzymes [141], or temperature [142] conditions, bioactive substances can be released from the film [143]. This mechanism not only improves the stability of bioactive substances but also enables their targeted release at the desired location based on environmental changes, thereby enhancing their bioavailability and functionality. The synthesis of COF-based hollow nanoparticles (h-NPs) exhibits good water dispersibility, high capacity, and thermal responsiveness for loading essential oil molecules, which aim to achieve longer-term preservation of fruits. Moreover, these h-NPs can be recycled and reused [142]. Some studies utilized molten-globule-state β-lactoglobulin nanoparticles (MG-BLGNPs) to encapsulate linalool (LN), in conjunction with a carboxymethyl chitosan (CMC) coating, to improve the shelf life of fresh-cut apples. The CMC coating demonstrated the most pronounced antibacterial activity, which can be linked to the increased loading capacity of MG-BLGNPs for LN, further enhanced by the greater unfolding of β-lactoglobulin (BLG) [144].

### 3.3. Loaded Active Substances

By analyzing examples of polysaccharide-based films loaded with active substances, we found that polysaccharide-based nanocomposite films using bioactive substances were shown to have improved the mechanical, barrier, antimicrobial, and antioxidant properties of polysaccharide-based edible nanocomposite films. Additionally, we found that the bioactive compounds added to the film mainly include essential oils, polyphenols, and a variety of biological extracts [145]. Essential oils consist of volatile compounds from plant parts with antimicrobial and antioxidant properties [146]. Certain essential oils are used for medicinal purposes, as an alternative to synthetic products, and as natural preservatives in food. Essential oils consist mainly of low-molecular-weight aliphatic and aromatic secondary metabolites [147,148], with terpenes (i.e., p-cymene, limonene, pinene, thymol, carvacrol, or menthol) and phenylpropenes (i.e., eugenol, cinnamaldehyde, or piperidine) being the major compounds [149]. For example, various bioactive substances were successfully loaded into polysaccharide-based nanocomposite films, and these composite films showed significant advantages in fruit preservation (in Table 2). They can not only effectively delay the decay of fruits but also maintain the nutrients and taste of fruits, thus providing an innovative solution to extend the freshness period of fruits.

## 4. Freshness Preservation Mechanism and Safety of Polysaccharide-Based Nanocomposite Films

It is well known that the main factors of fruit spoilage are oxidation and bacterial fungal infection, so the use of polysaccharide-based nanocomposite films with the antimicrobial and antioxidant properties of the fruit to protect the treatment can aid in fruit preservation. Polysaccharide-based nanocomposite films, comprising biologically active substances and a nano-framework structure, are incorporated into a homogeneous film exhibiting enhanced physicochemical properties [155]. Materials with good biocompatibility are uniformly dispersed in the polysaccharide matrix, and the tight entanglement and hydrogen bonding between the nanomaterials and the polysaccharide film contribute to the formation of a dense structure and give the film a nanoscale rough surface, increasing the contact area of the film to the fruit and the air in order to enhance its antimicrobial effect [156]. The different chemical properties of multiple materials have been utilized to achieve specific preservation purposes for different fruits. Some have been utilized due to lignin nanoparticles added to polymer matrices (e.g., starch and cellulose), which, due to the large number of aromatic structures contained in lignin, can effectively retard or prevent oxidant-induced oxidative processes, such as free radicals, for the purpose of improving the bioactivity and photostability of the film. Polydopamine (PDA) has excellent biocompatibility and can be spontaneously deposited on the surface of various organic or inorganic materials to provide UV-blocking ability. The bacterial concentration of LNP@PDA composite films was significantly lower than that of control and pure pectin films, suggesting that the growth of microorganisms was inhibited by LNP@PDA [157].

To prevent oxidation of fruits in the air, lysozyme nanofibers (LNFs) are used as an additive to synthesize branched starch (PL) composite films. The maximum uptake of DPPH scavenging activity of 15.0 wt% of LNFs (2.88 mg·cm^2^) was 76.7 ± 2.5. The higher antioxidant activity of the LNFs may be a result of the bioactive peptides exposed during nanofibrillarization as protein hydrolysis products and lysozyme-derived peptides have been reported to have higher free radical scavenging activity [158]. Polysaccharide-based nanocomposite films are more advanced materials for food packaging because of their high microbial inhibition efficiency, which can effectively inhibit the development of spoilage and extend the shelf life of food products.

### Safety of Polysaccharide-Based Nanofilms

Chemical preservatives have long been utilized to prolong the shelf life of food. However, the World Health Organization (WHO) recently prohibited using chemical preservatives for food preservation due to their detrimental effects on personal health and the environment [159,160]. In terms of the safety of polysaccharide-based nanocomposite films, selenium nanoparticles (SeNPs) have been modified using quaternized chitin (QC) and tea polyphenols (EGCG) (QC-EGCG-SeNPs). The degradation rate of the films also reached about 81% after 11 days. Thus, the prepared films showed good biodegradability. Healthy C57BL/6 mice were used to assess the biosafety the results showed that no death or poisoning of mice occurred in both experimental and control groups. During this period, the coat color, diet, water intake, locomotor activity, and excretion of the mice were without diarrhea. The safety of the film was proved [161,162]. The safety and efficacy of the film were successfully demonstrated.

Several experiments have confirmed the washability [163], degradability [164,165], and edibility [166] of polysaccharide-based films. Someone once developed dual-purpose composite coatings or films containing silk fibroin/cellulose nanocrystals (SF/CNCs). The bio-safety of SCA-CS was demonstrated by observing the effect of SCA-CS coating on the growth of hydroponic bean sprout seeds, which were passed through glycerol and natural aloe rhodopsin powder (AE) as bioactive agents. After 15 days of incubation, there was no significant difference in the mean plant height between the control group plant height (55.2 cm), plant height under SCA-CS supplemented with SF (1 mg/mL) (55.4 cm), and SCA-CS (58 cm) treatments [163]. The main barriers to the commercialization of polysaccharide-based nanocomposite films in the short term are still the toxic by-products produced during the synthesis of the materials and the time-consuming nature of the synthesis process, in particular, the lack of environmentally friendly reprocessing technologies for industrial-scale production [167].

## 5. Applications

Polysaccharide-based nanocomposite films, in addition to their utilization in the preservation of vegetables and fruit, are also of research value in the fields of medical adhesives and sealants due to their excellent antimicrobial, non-toxic, antioxidant, and biocompatible properties [168,169,170].

The main application area of polysaccharide-based nanocomposite films is still in food packaging. Electrospun nanofiber films loaded with clove extract effectively inhibited fungal growth (e.g., *Aspergillus niger* DDS7 and *Bacillus subtilis* DDS4) on samples [171]. Currently, the incorporation of bioactive compounds and nanomaterials into polysaccharide-based films not only enhances their antimicrobial and antioxidant properties but also improves their physical and chemical characteristics. These films leverage their mechanical strength and barrier properties for fruit preservation. Polysaccharide-based nanocomposite films have been applied to apples, bananas, mangoes, longans, lychees, and other fruits for fruit preservation and have demonstrated excellent physical and mechanical properties, as well as antimicrobial and antioxidant effects, to extend the shelf life of fruits during postharvest storage. In an experiment on the application of tea polyphenol-loaded sodium alginate and konjac glucomannan (TP-SA-KGM) for preserving apples, although the weight loss rate of apples preserved by TP-SA-KGM film was higher compared with the control polyethylene film, the total soluble sugar content of the control apples was significantly lower than that of apples wrapped in the polysaccharide-based film after 4 days of freshness preservation due to the strong inhibitory effect of the film on the respiration of the apples. This was due to the potent inhibitory effect of the film on the breathing of apples. Meanwhile, compared with the blank control group and the polyethylene film group, the diameter of apple wound rot in the TP-SA-KGM group was reduced by 35.61% and 8.62%, respectively, which proved that the TP-SA-KGM film could inhibit microbial activity and prolong the shelf-life of apples [172]. The electrostatically spun film exhibits elevated porosity, concomitant with augmented air permeability (WVP). The necessity for distinct WVP values for diverse fruits is well documented. However, the film’s reduced mechanical strength signifies potential for enhancement with respect to transportation [173]. Conversely, conventional fabrication methods offer distinct advantages, including higher mechanical properties, reduced cost, and mass production. However, these methods also present disadvantages, such as the tendency of nanomaterials or bioactives to aggregate on the surface of the film, which can hinder efficient loading of various materials [174].

**Figure 7:**
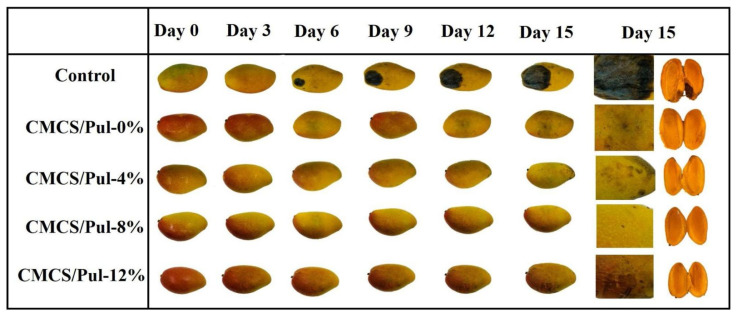
Application of CMCS/Pul-GEO blend film for fruit preservation [175].

In another mango preservation study, a functional edible film made from carboxymethyl chitosan (CMCS) and branched chain starch (Pul) infused with galangal essential oil (GEO) was developed. The blended films containing different GEOs were compared with a blank group. Blended films containing different GEO concentrations were compared with a control group. Changes in mango appearance during storage are shown in Figure 7. Obvious mold changes were observed in the blank control group after 6 d of storage at 25 °C. The results showed that CMCS/Pul films had better preservation properties relative to the control group. It is intuitively obvious that the CMCS/Pul-8% GEO film showed the best preservation results [175].

When packaging fruits for freshness, polysaccharide-based films can exhibit enhanced antioxidant properties and even improve the nutritional value of fruits over time. In a previous experiment, the addition of date nut powder to a pectin–chitosan composite film improved the physicochemical properties and biological activity of the pectin–chitosan composite film. The bioactivities of the prepared novel films, such as antioxidant and antimicrobial properties, enhanced performance with increasing concentration. Freshness preservation tests on grape berries showed that the films had a freshness preservation effect without affecting the quality of the berries. In addition, the release of bioactive compounds from the manufactured film into the grape berries not only prolonged the shelf-life but also significantly enhanced the phenolics of the grapes in the film over time compared to the control group [150].

In addition to conventional polysaccharide-based nanofilm packaging with freshness and antibacterial effects, more and more scientists are beginning to explore the polysaccharide-based film doped with polysaccharides that are sensitive to various types of stimuli and natural indicators that respond to external stimuli. For example, smart packaging systems designed to respond to pH changes [176,177], spoilage metabolites [178], temperature fluctuations [179], and gas composition [180] have been developed. Due to the respiration of fruits, the invasion of bacteria, and the mechanical damage of packaging, a series of biometabolites, such as ammonia [181], ethanol, and other metabolites, are accumulated inside the package. Smart packaging is mainly used to detect the freshness of fruits during storage and transportation [182]. Current research trends are centered on the environmentally friendly synthesis of nanoparticles, the optimization of multicomponent synergistic effects, and the development of smart indicator functions (e.g., color-responsive mechanisms of anthocyanin–silver nanoparticle composite films). However, there are still significant gaps in this field. Firstly, the long-term biosafety of nanocomposite films and the feasibility of large-scale production have not been systematically evaluated. Secondly, the influence of environmental conditions (temperature and humidity, microbial community) on the stability of film performance is not well studied. In addition, most experiments are limited to laboratory scale, and the preservation effect under different practical storage scenarios has not yet been fully analyzed. In the future, there is a need to explore the sustainable preparation process in combination with life cycle assessment (LCA) and reveal the molecular interactions between film materials and food matrices in order to promote the development of active packaging in the direction of high efficiency, safety, and intelligence [183].

## 6. Conclusions

**Figure 8:**
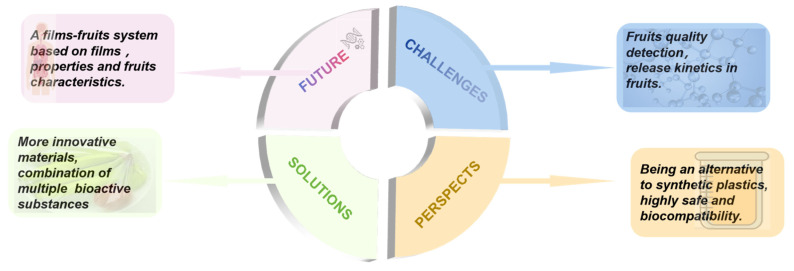
Several perspectives for future research.

Given the significant environmental issues associated with the buildup of fossil fuel-derived substances on Earth, the development of bio-based safety materials has garnered considerable attention. Polysaccharide-based nanocomposite films are widely used in various studies due to their availability and biocompatibility [139,184,185,186]. Films have significant potential for a variety of applications. Their drawbacks include poor water vapor barrier, mechanical properties, and low thermal stability. These drawbacks can be enhanced by incorporating various nanoparticles, such as silicon dioxide, silver, and cellulose nanoparticles, to improve their performance. Nanoparticles are commonly used as reinforcements in composites to enhance their mechanical and barrier properties [187]. Continued research into polysaccharides as nanocomposites is worthwhile for sustainable, useful, and cost-effective commodities. Fruit preservation technology is facing great challenges with the increasing consumer demand for fresh fruits, and polysaccharide-based nanocomposite films remain a major challenge as alternatives to synthetic food preservatives (e.g., potassium sorbate, sulfites, or nitrites) [188]. It represents an emerging paradigm that is defined as the reformulation of food products with fewer preservatives or none at all. The limited commercialization of polysaccharide-based films primarily arises from two factors: (1) elevated production costs compared to conventional PVC films and (2) insufficient mechanical and barrier performance. Current research suggests these challenges may be addressed through strategic material engineering: utilizing plant waste extracts to reduce raw material expenses and incorporating nanoscale reinforcements (e.g., cellulose nanofibers) to enhance functional properties. These biopolymer composites demonstrate unique advantages over petroleum-based alternatives, particularly through their inherent antimicrobial activity and potential for intelligent monitoring via responsive molecular design. The integration of dynamic crosslinking mechanisms (Schiff base interactions, metal coordination) enables the development of adaptive packaging systems that maintain structural integrity while offering active protection. Secondary issues such as production capacity and the evaluation of the economic cost of each reactive encapsulation technology still need to be addressed if they are to be put into production. Each fruit species is different, and susceptibility to bacterial fungi is also very different. We still need to study the kinetic growth models of fungal bacteria in different preserved fruits and using polysaccharide-based nanocomposite films. The effect of long-term consumption of polysaccharide-based nanocomposite films on the human body and whether polysaccharide-based nanocomposite films can penetrate the interior of fruit within a certain period still require further research (in Figure 8).

## Figures and Tables

**Table 1:** The timeline of polysaccharide-based nanocomposite films in the field of preservation of fruits.

Type of Films	Nanomaterials/Bioactive Substances	Date	Innovation	Reference
Chitosan films	Carvacrol nanoemulsions	2016	The addition of nanomaterials improves the homogeneity and surface hydrophobicity of the film	[62]
Gelatin films	Guar gum benzoate nanoparticles	2017	Superior barrier properties, reinforcing and thermal insulation effects	[63]
Chitosan films	TiO_2_ nanoparticles	2018	Ethylene photodegradation to prolong shelf life of fruit	[64]
Starch-PVA composite films	Zinc-oxide nanoparticles/phytochemicals	2019	The fruit in the film is intelligently monitored by the color of the film changes with the pH	[65]
Chitosan films	Nano-TiO_2_/litchi peel extract	2020	The activity of polyphenol oxidase, electrolyte leakage, and malondialdehyde accumulation were inhibited	[66]
κ-carrageenan and sodium carboxymethyl starch	Carboxylated cellulose nanocrystals	2021	This material resists shrinking effectively and maintains durability even with regular use	[67]
Carboxymethyl starch films	MOF-199/curcumin	2022	The nanomaterial is decomposed by water, and curcumin is released to prolong shelf life	[68]
Chitosan sodium alginate composite films	ZIF-8/quantum dots	2023	Efficient sterilization under light conditions and ethylene removal effect	[69]
Alginate films	Nanometer-calcium	2024	Bodegradable, pH-triggered hydrogel films promoted enhanced polyphenol–calcium binding	[70]

**Table 2:** Overview of polysaccharide-based films loaded with bioactive compounds for fruit applications.

Bioactive Substance	Types of Films	Applied Fresh Food Types	Apply Effect	Reference
Jujube Seed Powder	Pectin–chitosan composite film	Grapes	No browning effect was observed in the grapes kept in the films during 10 days.	[150]
Curcumin	Chitosan-based films	Lychee, Strawberry, Mango and Plum	After 9 days, there was no mold growth on the outside of the samples and the pulp was still shiny and translucent.	[151]
Propolis Extract	Pectin-based films	Strawberry	Showed a lower rate of spoilage from day 3 onwards.	[152]
Deep Eutectic Solvent Extraction of Tomato Extract	Chitosan film	Strawberry	Within 9 days, strawberry rot was reduced by 55.41%.	[65]
Tannins and Cinnamaldehyde	Chitosan film	Mandarin Orange (Citrus Reticulata)	Removes more than 99.9% of bacteria and fungi, significantly extending the shelf life of citrus by approximately 1.9 times.	[153]
Ginkgo Biloba Extract	Chitosan film	Banana and Cherry Tomato	The treated banana and cherry tomatoes showed better sensory quality on day 8 and 20, respectively.	[154]

## Data Availability

No new data were created or analyzed in this study. Data sharing is not applicable to this article.

## References

[1] Zhao P., Ndayambaje J.P., Liu X., Xia X. (2020). Microbial Spoilage of Fruits: A Review on Causes and Prevention Methods. Food Rev. Int..

[2] Priyadarshi R., Jayakumar A., de Souza C.K., Rhim J.W., Kim J.T. (2024). Advances in strawberry postharvest preservation and packaging: A comprehensive review. Compr. Rev. Food Sci. Food Saf..

[3] Pal K., Sarkar P., Anis A., Wiszumirska K., Jarzębski M. (2021). Polysaccharide-Based Nanocomposites for Food Packaging Applications. Materials.

[4] Bukhari N.T.M., Rawi N.F.M., Hassan N.A.A., Saharudin N.I., Kassim M.H.M. (2023). Seaweed polysaccharide nanocomposite films: A review. Int. J. Biol. Macromol..

[5] Sahana T.G., Rekha P.D. (2018). Biopolymers: Applications in wound healing and skin tissue engineering. Mol. Biol. Rep..

[6] Liu Y., Liu J., Song P. (2021). Recent advances in polysaccharide-based carbon aerogels for environmental remediation and sustainable energy. Sustain. Mater. Technol..

[7] Su M., Liu F., Abdiryim T., Liu X. (2024). Polysaccharides and their derivatives for solar-driven water evaporators. Cellulose.

[8] Shi J., Xiao Y., Fu G., Feng C., Hu J., Haegeman W., Liu H. (2023). Calcareous silt earthen construction using biopolymer reinforcement. J. Build. Eng..

[9] Wang Y., Li R., Lu R., Xu J., Hu K., Liu Y. (2019). Preparation of Chitosan/Corn Starch/Cinnamaldehyde Films for Strawberry Preservation. Foods.

[10] Xue C., Linqin C., Xue Y., Yilin W., Jiaxin X., Rongfan Z., Shaole L., Yuanyuan L. (2024). Active curcumin-loaded γ-cyclodextrin-metal organic frameworks as nano respiratory channels for reinforcing chitosan/gelatin films in strawberry preservation. Food Hydrocoll..

[11] Nadar S.S., Vaidya L., Maurya S., Rathod V.K. (2019). Polysaccharide based metal organic frameworks (polysaccharide–MOF): A review. Coord. Chem. Rev..

[12] Li Y., Bian B., Tang R., Zhang K. (2024). Characterization of a Clove Essential Oil Slow-Release Microencapsulated Composite Film and Its Preservation Effects on Blueberry. ACS Omega.

[13] Geng C., Jiang Y., Bian H., Huang G. (2024). Selective gas-permeation films with nanoMOFs as gas “Switches” for mango preservation. Chem. Eng. J..

[14] Xu M., Fang D., Shi C., Xia S., Wang J., Deng B., Kimatu B.M., Guo Y., Lyu L., Wu Y. (2024). Anthocyanin-loaded polylactic acid/quaternized chitosan electrospun nanofiber as an intelligent and active packaging film in blueberry preservation. Food Hydrocoll..

[15] Kim S.-H., Kang S.E., Kim Y.-D., Park M.-K. (2024). Industrial-scale blown active packaging film with essential oils: Properties, dual-functional performance, and box packaging application of instant noodles. Food Packag. Shelf Life.

[16] Wu X., Gong D., Zhao K., Chen D., Dong Y., Gao Y., Wang Q., Hao G.-F. (2024). Research and development trends in plant growth regulators. Adv. Agrochem..

[17] Li B.-J., Grierson D., Shi Y., Chen K.-S. (2022). Roles of abscisic acid in regulating ripening and quality of strawberry, a model non-climacteric fruit. Hortic. Res..

[18] Martín-Pizarro C., Vallarino J.G., Osorio S., Meco V., Urrutia M., Pillet J., Casañal A., Merchante C., Amaya I., Willmitzer L. (2021). The NAC transcription factor FaRIF controls fruit ripening in strawberry. Plant Cell.

[19] Zhao H., Zhang S., Ma D., Liu Z., Qi P., Wang Z., Di S., Wang X. (2024). Review of fruits flavor deterioration in postharvest storage: Odorants, formation mechanism and quality control. Food Res. Int..

[20] An X., Zhu P., Li Z., Fadiji T., Wani A.A. (2023). Effect of expanded polyethylene (EPE) foam packing net design on the mechanical damage resistance of strawberry fruit during transportation. Food Packag. Shelf Life.

[21] Wilson M.D., Stanley R.A., Eyles A., Ross T. (2017). Innovative processes and technologies for modified atmosphere packaging of fresh and fresh-cut fruits and vegetables. Crit. Rev. Food Sci. Nutr..

[22] Yan Y., Zhang Y., Fang Z., Wang Z.-C., Nan Y., Shi H., Zhang H., Song W., Gu H. (2024). Modified atmosphere packaging and plant extracts synergistically enhance the preservation of meat: A review. Food Control.

[23] Bisht B., Bhatnagar P., Gururani P., Kumar V., Tomar M.S., Sinhmar R., Rathi N., Kumar S. (2021). Food irradiation: Effect of ionizing and non-ionizing radiations on preservation of fruits and vegetables—A review. Trends Food Sci. Technol..

[24] Assumpção C.F., Hermes V.S., Pagno C., Castagna A., Mannucci A., Sgherri C., Pinzino C., Ranieri A., Flôres S.H., Rios A.d.O. (2018). Phenolic enrichment in apple skin following post-harvest fruit UV-B treatment. Postharvest Biol. Technol..

[25] Pellicer J.A., Gabaldón J.A., Gómez-López V.M. (2021). Effect of pH on pulsed light inactivation of polyphenol oxidase. Enzym. Microb. Technol..

[26] Salazar-Zúñiga M.N., Lugo-Cervantes E., Rodríguez-Campos J., Sanchez-Vega R., Rodríguez-Roque M.J., Valdivia-Nájar C.G. (2022). Pulsed Light Processing in the Preservation of Juices and Fresh-Cut Fruits: A Review. Food Bioprocess Technol..

[27] Cheigh C.-I., Hwang H.-J., Chung M.-S. (2013). Intense pulsed light (IPL) and UV-C treatments for inactivating Listeria monocytogenes on solid medium and seafoods. Food Res. Int..

[28] Gyawali R., Degala H.L., Biswal A.K., Bardsley C.A., Mahapatra A.K. (2024). Effects of intense pulsed light on inactivation of Salmonella Typhimurium and quality characteristics of pecan halves. LWT—Food Sci. Technol..

[29] Kim H.-J., Jubinville E., Goulet-Beaulieu V., Jean J. (2024). Inactivation of murine norovirus and hepatitis A virus on various frozen fruits using pulsed light. Int. J. Food Microbiol..

[30] Karaoglan H.A., Keklik N.M., Develi Işikli N. (2016). Modeling Inactivation ofCandida inconspicuaIsolated from Turnip Juice using Pulsed UV Light. J. Food Process Eng..

[31] Oyom W., Zhang Z., Bi Y., Tahergorabi R. (2022). Application of starch-based coatings incorporated with antimicrobial agents for preservation of fruits and vegetables: A review. Prog. Org. Coat..

[32] Zhu Y., Li D., Belwal T., Li L., Chen H., Xu T., Luo Z. (2019). Effect of Nano-SiOx/Chitosan Complex Coating on the Physicochemical Characteristics and Preservation Performance of Green Tomato. Molecules.

[33] Du Y., Shi B., Luan X., Wang Y., Song H. (2023). Chitosan/cellulose nanocrystal biocomposite coating for fruit postharvest preservation. Ind. Crops Prod..

[34] Wu J., Zhang J., Ni W., Xu X., George M.S., Lu G. (2023). Effect of Heat Treatment on the Quality and Soft Rot Resistance of Sweet Potato during Long-Term Storage. Foods.

[35] Li Y., Hua Z., Li Y., Chen T., Alamri A.S., Xu Y., Gong W., Hou Y., Alhomrani M., Hu J. (2024). Development of multifunctional chitosan-based composite film loaded with tea polyphenol nanoparticles for strawberry preservation. Int. J. Biol. Macromol..

[36] Tang J., Huang C., Liu W., Zeng X., Zhang J., Liu W., Pang J., Wu C. (2024). Preparation and characterization of a konjac glucomannan-based bio-nanocomposite film and its application in cherry tomato preservation. Food Hydrocoll..

[37] Wang S., Ma Y., Wang F., Lu C., Liu Y., Zhang S., Ma S., Wang L. (2025). Development of cellulose-based self-healing hydrogel smart packaging for fish preservation and freshness indication. Carbohydr. Polym..

[38] Yin C., Ding X., Lin Z., Cao J., Shi W., Wang J., Xu D., Xu D., Liu Y., Liu G. (2024). Preparation and characterization of quercetin@ZIF-L/GO@AgNPs nanocomposite film for room-temperature strawberry preservation. Food Chem..

[39] Singh R., Dutt S., Sharma P., Sundramoorthy A.K., Dubey A., Singh A., Arya S. (2023). Future of Nanotechnology in Food Industry: Challenges in Processing, Packaging, and Food Safety. Glob. Chall..

[40] Long J., Zhang W., Zhao M., Ruan C.-Q. (2023). The reduce of water vapor permeability of polysaccharide-based films in food packaging: A comprehensive review. Carbohydr. Polym..

[41] Khan A., Khan R.A., Salmieri S., Le Tien C., Riedl B., Bouchard J., Chauve G., Tan V., Kamal M.R., Lacroix M. (2012). Mechanical and barrier properties of nanocrystalline cellulose reinforced chitosan based nanocomposite films. Carbohydr. Polym..

[42] Sun J., Chen R., Zhang S., Bai Y., Zhao P., Zhou H., Long M., Wang X., Meng Y.H., Guo Y. (2024). Pectin-containing lignocellulosic nanofibers isolated from young apples enhance chitosan preservation film with robust mechanical and barrier properties. Food Hydrocoll..

[43] Ying Ben Z., Samsudin H., Firdaus Yhaya M. (2022). Glycerol: Its properties, polymer synthesis, and applications in starch based films. Eur. Polym. J..

[44] Zhang W., Roy S., Assadpour E., Cong X., Jafari S.M. (2023). Cross-linked biopolymeric films by citric acid for food packaging and preservation. Adv. Colloid Interface Sci..

[45] Shao H., Sun H., Yang B., Zhang H., Hu Y. (2019). Facile and green preparation of hemicellulose-based film with elevated hydrophobicity via cross-linking with citric acid. RSC Adv..

[46] Li J., Li M., Wang Z., Hu G., Yang W., Hu Y. (2024). Chitosan/PCL fibrous membranes loaded potassium cinnamate/β-CD clathrate compounds developed by ELS for fruit preservation. Food Hydrocoll..

[47] Lai W., Wang L., Pang Y., Xin M., Li M., Shi L., Mao Y. (2024). Preparation of citric acid cross-linked chitosan quaternary phosphonium/polyvinyl alcohol composite film and its application in strawberry preservation. Food Chem..

[48] Qin Z., Huang Y., Xiao S., Zhang H., Lu Y., Xu K. (2022). Preparation and Characterization of High Mechanical Strength Chitosan/Oxidized Tannic Acid Composite Film with Schiff Base and Hydrogen Bond Crosslinking. Int. J. Mol. Sci..

[49] Zhao Y., Yao G., Li K., Ye J., Chen J., Zhang J. (2024). Preparation, characterization, and antibacterial application of cross-linked nanoparticles composite films. Food Chem. X.

[50] Qiao J., Wang Q., Liu K., Chang Y., Wang L., Zhang S., Yu Y. (2024). Characterization and Antioxidant and Antibacterial Activities of Carboxymethylated Tamarind Seed Polysaccharide Composite Films Incorporated with ε-Polylysine and Their Application in Fresh-Cut Green Bell Pepper Preservation. J. Agric. Food Chem..

[51] Zhang X., Li Z., Ji R., Li K., Zhang W. (2021). Preparation and Characterization of Pullulan/Carboxymethyl Cellulose/Nano-TiO_2_ Composite Films for Strawberry Preservation. Food Biophys..

[52] Yu H.C., Zhang H., Ren K., Ying Z., Zhu F., Qian J., Ji J., Wu Z.L., Zheng Q. (2018). Ultrathin κ-Carrageenan/Chitosan Hydrogel Films with High Toughness and Antiadhesion Property. ACS Appl. Mater. Interfaces.

[53] Chen H., Hu X., Chen E., Wu S., McClements D.J., Liu S., Li B., Li Y. (2016). Preparation, characterization, and properties of chitosan films with cinnamaldehyde nanoemulsions. Food Hydrocoll..

[54] Dai Q., Huang X., Jia R., Fang Y., Qin Z. (2022). Development of antibacterial film based on alginate fiber, and peanut red skin extract for food packaging. J. Food Eng..

[55] Meng Y., Zhao H., Dong C., He Z., Long Z. (2024). Eco-friendly and flexible polysaccharide-based packaging films for fruit preservation. Int. J. Biol. Macromol..

[56] Ferreira R.R., Souza A.G., Quispe Y.M., Rosa D.S. (2021). Essential oils loaded-chitosan nanocapsules incorporation in biodegradable starch films: A strategy to improve fruits shelf life. Int. J. Biol. Macromol..

[57] Li J., Zhou M., Cheng G., Cheng F., Lin Y., Zhu P.-X. (2019). Fabrication and characterization of starch-based nanocomposites reinforced with montmorillonite and cellulose nanofibers. Carbohydr. Polym..

[58] Meira S.M.M., Zehetmeyer G., Werner J.O., Brandelli A. (2017). A novel active packaging material based on starch-halloysite nanocomposites incorporating antimicrobial peptides. Food Hydrocoll..

[59] Zhang Y., Zhang M., Yang H. (2015). Postharvest chitosan-g-salicylic acid application alleviates chilling injury and preserves cucumber fruit quality during cold storage. Food Chem..

[60] Zhao K., Wang W., Teng A., Zhang K., Ma Y., Duan S., Li S., Guo Y. (2020). Using cellulose nanofibers to reinforce polysaccharide films: Blending vs layer-by-layer casting. Carbohydr. Polym..

[61] Tang H., Han Z., Zhao C., Jiang Q., Tang Y., Li Y., Cheng Z. (2023). Preparation and characterization of Aloe vera polysaccharide-based packaging film and its application in blueberry preservation. Prog. Org. Coat..

[62] Tastan Ö., Ferrari G., Baysal T., Donsì F. (2016). Understanding the effect of formulation on functionality of modified chitosan films containing carvacrol nanoemulsions. Food Hydrocoll..

[63] Kundu S., Das A., Basu A., Abdullah M.F., Mukherjee A. (2017). Guar gum benzoate nanoparticle reinforced gelatin films for enhanced thermal insulation, mechanical and antimicrobial properties. Carbohydr. Polym..

[64] Kaewklin P., Siripatrawan U., Suwanagul A., Lee Y.S. (2018). Active packaging from chitosan-titanium dioxide nanocomposite film for prolonging storage life of tomato fruit. Int. J. Biol. Macromol..

[65] Yu J., Xu S., Chen R., Shao P. (2024). A promising bioactive chitosan film in strawberry fresh-keeping: Plasticized with tomato processing by-product extract of deep eutectic solvent. Food Hydrocoll..

[66] Liu Z., Du M., Liu H., Zhang K., Xu X., Liu K., Tu J., Liu Q. (2020). Chitosan films incorporating litchi peel extract and titanium dioxide nanoparticles and their application as coatings on watercored apples. Prog. Org. Coat..

[67] Zhang C., Chi W., Meng F., Wang L. (2021). Fabricating an anti-shrinking κ-carrageenan/sodium carboxymethyl starch film by incorporating carboxylated cellulose nanofibrils for fruit preservation. Int. J. Biol. Macromol..

[68] Liang Y., Yao Y., Liu Y., Li Y., Xu C., Fu L., Lin B. (2022). Curcumin-loaded HKUST-1@ carboxymethyl starch-based composites with moisture-responsive release properties and synergistic antibacterial effect for perishable fruits. Int. J. Biol. Macromol..

[69] Wang M., Nian L., Wu J., Cheng S., Yang Z., Cao C. (2023). Visible light-responsive chitosan/sodium alginate/QDs@ZIF-8 nanocomposite films with antibacterial and ethylene scavenging performance for kiwifruit preservation. Food Hydrocoll..

[70] Zhang K., Zhang W., Kong Y., Wang S., Yao B., Wang Y., Wang Z. (2024). Truxillic calcium supramolecular skeleton fortified pH responsive and biodegradable alginate hydrogel films promoting fruit preservation. Int. J. Biol. Macromol..

[71] Wang Y., Ju J., Diao Y., Zhao F., Yang Q. (2024). The application of starch-based edible film in food preservation: A comprehensive review. Crit. Rev. Food Sci. Nutr..

[72] Xin S., Xiao L., Dong X., Li X., Wang Y., Hu X., Sameen D.E., Qin W., Zhu B. (2020). Preparation of chitosan/curcumin nanoparticles based zein and potato starch composite films for Schizothorax prenati fillet preservation. Int. J. Biol. Macromol..

[73] Balakrishnan P., Gopi S., Sreekala M.S., Thomas S. (2017). UV resistant transparent bionanocomposite films based on potato starch/cellulose for sustainable packaging. Starch.

[74] Oleyaei S.A., Almasi H., Ghanbarzadeh B., Moayedi A.A. (2016). Synergistic reinforcing effect of TiO_2_ and montmorillonite on potato starch nanocomposite films: Thermal, mechanical and barrier properties. Carbohydr. Polym..

[75] Ali A., Xie F., Yu L., Liu H., Meng L., Khalid S., Chen L. (2018). Preparation and characterization of starch-based composite films reinfoced by polysaccharide-based crystals. Compos. Part B Eng..

[76] Li H., Wang J., Liu Y., Chen J., Wang C., Hu Y., Hu K. (2024). Production of biodegradable potato starch films containing Lycium barbarum polysaccharide and investigation of their physicochemical properties. Food Packag. Shelf Life.

[77] Ma Y., Zhao H., Ma Q., Cheng D., Zhang Y., Wang W., Wang J., Sun J. (2022). Development of chitosan/potato peel polyphenols nanoparticles driven extended-release antioxidant films based on potato starch. Food Packag. Shelf Life.

[78] Yue Z., Ziheng L., Jie Z., Haiyan G., Junru Q. (2025). Barrier properties characterization and release kinetics study of citrus fibers-reinforced functional starch composites. Food Hydrocoll..

[79] Chen K., Tian R., Jiang J., Xiao M., Wu K., Kuang Y., Deng P., Zhao X., Jiang F. (2024). Moisture loss inhibition with biopolymer films for preservation of fruits and vegetables: A review. Int. J. Biol. Macromol..

[80] Vargas-Torrico M.F., von Borries-Medrano E., Aguilar-Méndez M.A. (2022). Development of gelatin/carboxymethylcellulose active films containing Hass avocado peel extract and their application as a packaging for the preservation of berries. Int. J. Biol. Macromol..

[81] Liu M., Wang Y., Su S., Long F., Zhong L., Hu J. (2024). Multifunctional bio-nanocomposite films integrated with essential oils@metal-phenolic network nanocapsules for durable fruit preservation. Int. J. Biol. Macromol..

[82] Nongnual T., Butprom N., Boonsang S., Kaewpirom S. (2024). Citric acid crosslinked carboxymethyl cellulose edible films: A case study on preserving freshness in bananas. Int. J. Biol. Macromol..

[83] Lakshmi D.S., Trivedi N., Reddy C.R.K. (2017). Synthesis and characterization of seaweed cellulose derived carboxymethyl cellulose. Carbohydr. Polym..

[84] Azarifar M., Ghanbarzadeh B., Sowti Khiabani M., Akhondzadeh Basti A., Abdulkhani A., Noshirvani N., Hosseini M. (2019). The optimization of gelatin-CMC based active films containing chitin nanofiber and Trachyspermum ammi essential oil by Response Surface Methodology. Carbohydr. Polym..

[85] Shen Y., Seidi F., Ahmad M., Liu Y., Saeb M.R., Akbari A., Xiao H. (2023). Recent Advances in Functional Cellulose-based Films with Antimicrobial and Antioxidant Properties for Food Packaging. J. Agric. Food Chem..

[86] Chen Y., Li Y., Qin S., Han S., Qi H. (2022). Antimicrobial, UV blocking, water-resistant and degradable coatings and packaging films based on wheat gluten and lignocellulose for food preservation. Compos. Part B Eng..

[87] Sun J., Yang X., Bai Y., Fang Z., Zhang S., Wang X., Yang Y., Guo Y. (2024). Recent Advances in Cellulose Nanofiber Modification and Characterization and Cellulose Nanofiber-Based Films for Eco-Friendly Active Food Packaging. Foods.

[88] Sanchez-Salvador J.L., Xu H., Balea A., Blanco A., Negro C. (2024). Enhancement of the production of TEMPO-mediated oxidation cellulose nanofibrils by kneading. Int. J. Biol. Macromol..

[89] Zhang Q., Yang W., Zhang S., Tang J., Shi X., Qin S., Pan L., Xiao H. (2023). Enhancing the applicability of gelatin-carboxymethyl cellulose films by cold plasma modification for the preservation of fruits. LWT—Food Sci. Technol..

[90] Cheng L., Zhang D., Gu Z., Li Z., Hong Y., Li C. (2018). Preparation of acetylated nanofibrillated cellulose from corn stalk microcrystalline cellulose and its reinforcing effect on starch films. Int. J. Biol. Macromol..

[91] Shen H., Chen J., Tan K.B. (2024). Ethyl cellulose matrixed poly(sulfur-co-sorbic acid) composite films: Regulation of properties and application for food preservation. Int. J. Biol. Macromol..

[92] Zanini M., Lavoratti A., Lazzari L.K., Galiotto D., Pagnocelli M., Baldasso C., Zattera A.J. (2016). Producing aerogels from silanized cellulose nanofiber suspension. Cellulose.

[93] Yao F., Wu Z., Gu Y., Di Y., Liu Y., Srinivasan V., Lian C., Li Y. (2024). Acetylated nanocellulose reinforced hydroxypropyl starch acetate realizing polypropylene replacement for green packaging application. Carbohydr. Polym..

[94] Kumar S., Mukherjee A., Dutta J. (2020). Chitosan based nanocomposite films and coatings: Emerging antimicrobial food packaging alternatives. Trends Food Sci. Technol..

[95] Zhou X., Liu X., Wang Q., Lin G., Yang H., Yu D., Cui S.W., Xia W. (2022). Antimicrobial and antioxidant films formed by bacterial cellulose, chitosan and tea polyphenol—Shelf life extension of grass carp. Food Packag. Shelf Life.

[96] Ding K., Xie Y., Xu H., Xu S., Ge S., Li H., Chang X., Chen J., Wang R., Shan Y. (2024). Visible light-responsive TiO_2_-based hybrid nanofiller reinforced multifunctional chitosan film for effective fruit preservation. Food Chem..

[97] Chen K., Brennan C., Cao J., Cheng G., Li L., Qin Y., Chen H. (2023). Characterization of chitosan/eugenol-loaded IRMOF-3 nanoparticles composite films with sustained antibacterial activity and their application in postharvest preservation of strawberries. LWT—Food Sci. Technol..

[98] Ji Q., Jin Z., Ding W., Wu Y., Liu C., Yu K., Zhang N., Jin G., Lu P., Bao D. (2023). Chitosan composite films based on tea seed oil nano-microcapsules: Antibacterial, antioxidant and physicochemical properties. Food Packag. Shelf Life.

[99] Fu H., Huang R., Li J., Lin Z., Wei F., Lin B. (2023). Multifunctional cinnamaldehyde-tannic acid nano-emulsion/chitosan composite film for mushroom preservation. Food Hydrocoll..

[100] Khan S., Hashim S.B.H., Arslan M., Zhang K., Siman L., Mukhtar A., Zhihua L., Tahir H.E., Zhai X., Shishir M.R.I. (2024). Development of an active biogenic silver nanoparticles composite film based on berry wax and chitosan for rabbit meat preservation. Int. J. Biol. Macromol..

[101] Kerch G. (2015). Chitosan films and coatings prevent losses of fresh fruit nutritional quality: A review. Trends Food Sci. Technol..

[102] Cho Rok L., Su Jin L., Tae In K., Kiramage C., Jong Soo L., Sangsik K., Min Hee K., Won Ho P. (2024). Chitosan-gallic acid conjugate edible coating film for perishable fruits. Food Chem..

[103] Roy S., Priyadarshi R., Rhim J.-W. (2021). Development of Multifunctional Pullulan/Chitosan-Based Composite Films Reinforced with ZnO Nanoparticles and Propolis for Meat Packaging Applications. Foods.

[104] Wu J., Zhang Y., Zhang F., Mi S., Yu W., Sang Y., Wang X. (2024). Preparation of chitosan/polyvinyl alcohol antibacterial indicator composite film loaded with AgNPs and purple sweet potato anthocyanins and its application in strawberry preservation. Food Chem..

[105] Liu Z., Wang S., Liang H., Zhou J., Zong M., Cao Y., Lou W. (2024). A review of advancements in chitosan-essential oil composite films: Better and sustainable food preservation with biodegradable packaging. Int. J. Biol. Macromol..

[106] Nesren M.E.-B., Soliman M.A.S., Neveen M.K., Mohamed N.A.E.-G. (2025). Chitosan and alginate/Aspergillus flavus-mediated nanocomposite films for preservation of postharvest tomatoe. Int. J. Biol. Macromol..

[107] Wang Y., Zhang Y., Ma Y., Liu J., Zhang R. (2025). Preparation and application of chitosan/nano-TiO_2_/daisy essential oil composite films in the preservation of Actinidia arguta. Food Chem. X.

[108] Zhang X., Li G., Chen C., Fan H., Fang J., Wu X., Qi J., Li H. (2025). Chitosan/PVA composite film enhanced by ZnO/lignin with high-strength and antibacterial properties for food packaging. Int. J. Biol. Macromol..

[109] He J., Zhang W., Goksen G., Khan M.R., Ahmad N., Cong X. (2024). Functionalized sodium alginate composite films based on double-encapsulated essential oil of wampee nanoparticles: A green preservation material. Food Chem. X.

[110] Han Y., Zhou M., Deng J., Cheng C., Xu Z., Hou W., Yi Y., McClements D.J., Chen S. (2024). Intelligent carrageenan-based composite films containing color indicator-loaded nanoparticles for monitoring fish freshness. Food Packag. Shelf Life.

[111] Orsuwan A., Shankar S., Wang L.-F., Sothornvit R., Rhim J.-W. (2016). Preparation of antimicrobial agar/banana powder blend films reinforced with silver nanoparticles. Food Hydrocoll..

[112] Krishnan L., Ravi N., Kumar Mondal A., Akter F., Kumar M., Ralph P., Kuzhiumparambil U. (2024). Seaweed-based polysaccharides—Review of extraction, characterization, and bioplastic application. Green Chem..

[113] Giriyappa Thimmaiah P., Mudinepalli V.R., Thota S.R., Obireddy S.R., Lai W.-F. (2022). Preparation, Characterization and Dielectric Properties of Alginate-Based Composite Films Containing Lithium Silver Oxide Nanoparticles. Front. Chem..

[114] Li S., Hu X., Zhang S., Zhao J., Wang R., Wang L., Wang X., Yuan Y., Yue T., Cai R. (2024). A versatile bilayer smart packaging based on konjac glucomannan/alginate for maintaining and monitoring seafood freshness. Carbohydr. Polym..

[115] Udo T., Mummaleti G., Mohan A., Singh R.K., Kong F. (2023). Current and emerging applications of carrageenan in the food industry. Food Res. Int..

[116] Mostafavi F.S., Zaeim D. (2020). Agar-based edible films for food packaging applications—A review. Int. J. Biol. Macromol..

[117] Yang Z., Zhai X., Zhang C., Shi J., Huang X., Li Z., Zou X., Gong Y., Holmes M., Povey M. (2021). Agar/TiO_2_/radish anthocyanin/neem essential oil bionanocomposite bilayer films with improved bioactive capability and electrochemical writing property for banana preservation. Food Hydrocoll..

[118] Nguyen T.T., Huynh Nguyen T.-T., Tran Pham B.-T., Van Tran T., Bach L.G., Bui Thi P.Q., Ha Thuc C.N. (2021). Development of poly (vinyl alcohol)/agar/maltodextrin coating containing silver nanoparticles for banana (*Musa acuminate*) preservation. Food Packag. Shelf Life.

[119] Sun S., Wang N., Ali E., Qiao L., Guo Q., Lu L. (2024). Glucose-responsive carboxymethyl chitosan/ sodium alginate film protects against mechanical wound on postharvest blueberry. Food Hydrocoll..

[120] Yang Z., Zhai X., Li M., Li Z., Shi J., Huang X., Zou X., Yan M., Qian W., Gong Y. (2022). Saccharomyces cerevisiae-incorporated and sucrose-rich sodium alginate film: An effective antioxidant packaging film for longan preservation. Int. J. Biol. Macromol..

[121] Kang L., Liang Q., Rashid A., Qayum A., Chi Z., Ren X., Ma H. (2022). Ultrasound-assisted development and characterization of novel polyphenol-loaded pullulan/trehalose composite films for fruit preservation. Ultrason. Sonochem..

[122] Veiga-Santos P., Oliveira L.M., Cereda M.P., Scamparini A.R.P. (2007). Sucrose and inverted sugar as plasticizer. Effect on cassava starch–gelatin film mechanical properties, hydrophilicity and water activity. Food Chem..

[123] Moura-Alves M., Souza V.G.L., Silva J.A., Esteves A., Pastrana L.M., Saraiva C., Cerqueira M.A. (2023). Characterization of Sodium Alginate-Based Films Blended with Olive Leaf and Laurel Leaf Extracts Obtained by Ultrasound-Assisted Technology. Foods.

[124] Chen J., Zhang Y., Liu H., Lu H., Xu X., Shen M. (2024). Sodium alginate-camellia seed cake protein active packaging film cross-linked by electrostatic interactions for fruit preservation. Int. J. Biol. Macromol..

[125] Ruchika, Sharma S.K., Kumar R., Yadav S.K., Saneja A. (2024). Development of a multifunctional and sustainable pterostilbene nanoemulsion incorporated chitosan-alginate food packaging film for shiitake mushroom preservation. Int. J. Biol. Macromol..

[126] Xing Y., Li W., Wang Q., Li X., Xu Q., Guo X., Bi X., Liu X., Shui Y., Lin H. (2019). Antimicrobial Nanoparticles Incorporated in Edible Coatings and Films for the Preservation of Fruits and Vegetables. Molecules.

[127] Zhang W., Sani M.A., Zhang Z., McClements D.J., Jafari S.M. (2023). High performance biopolymeric packaging films containing zinc oxide nanoparticles for fresh food preservation: A review. Int. J. Biol. Macromol..

[128] Wang G., Yang X., Chen X., Huang J., He R., Zhang R., Zhang Y. (2024). Construction and antibacterial activities of walnut green husk polysaccharide based silver nanoparticles (AgNPs). Int. J. Biol. Macromol..

[129] Liu J., Liu C., Zheng X., Chen M., Tang K. (2020). Soluble soybean polysaccharide/nano zinc oxide antimicrobial nanocomposite films reinforced with microfibrillated cellulose. Int. J. Biol. Macromol..

[130] Xu H., Quan Q., Chang X., Ge S., Xu S., Wang R., Xu Y., Luo Z., Shan Y., Ding S. (2023). A new nanohybrid particle reinforced multifunctional active packaging film for efficiently preserving postharvest fruit. Food Hydrocoll..

[131] Nawaz A.H., Mehmood A., Khan M.A., Ahmad K.S., Nabi A.G. (2024). Green synthesis of silver nanoparticles for their antifungal activity against anthracnose disease causing Colletotrichumcapsici. Biocatal. Agric. Biotechnol..

[132] Ton-That P., Dinh T.A., Thanh Gia-Thien H., Van Minh N., Nguyen T., Huynh K.P.H. (2024). Novel packaging chitosan film decorated with green-synthesized nanosilver derived from dragon fruit stem. Food Hydrocoll..

[133] Xiang F., Xia Y., Wang Y., Wang Y., Wu K., Ni X. (2021). Preparation of konjac glucomannan based films reinforced with nanoparticles and its effect on cherry tomatoes preservation. Food Packag. Shelf Life.

[134] Surendhiran D., Roy V.C., Park J.-S., Chun B.-S. (2022). Fabrication of chitosan-based food packaging film impregnated with turmeric essential oil (TEO)-loaded magnetic-silica nanocomposites for surimi preservation. Int. J. Biol. Macromol..

[135] Rodríguez-González C., Martínez-Hernández A.L., Castaño V.M., Kharissova O.V., Ruoff R.S., Velasco-Santos C. (2012). Polysaccharide Nanocomposites Reinforced with Graphene Oxide and Keratin-Grafted Graphene Oxide. Ind. Eng. Chem. Res..

[136] Dat N.M., Nam N.T.H., Cong C.Q., Huong L.M., Hai N.D., Tai L.T., An H., Duy B.T., Dat N.T., Viet V.N.D. (2023). Chitosan membrane drafting silver-immobilized graphene oxide nanocomposite for banana preservation: Fabrication, physicochemical properties, bioactivities, and application. Int. J. Biol. Macromol..

[137] Ma X., Zhao X., Zhang N., Liu X., Ding J., Xu Q., Cui K., Wang Y., Zhang Q., Cong H. (2024). Novel graphene oxide modified LDH@CEO antibacterial materials capable of NIR stimuli-responsive control release for food preservation. Colloids Surf. A Physicochem. Eng. Asp..

[138] Zhao J., Yang H., Li C., Xu Z., Shan P., Fu C., Tao Y., Hu J., Wang H., Du J. (2025). Eco-friendly chitosan-based composite film with anti-dissolution capacity as active packaging for fruit preservation. Food Hydrocoll..

[139] Yang W., Zhang S., Li C., Li M., Li Y., Wu P., Li H., Ai S. (2024). Functionalized cellulose nanocrystals reinforced polysaccharide matrix for the preparation of active packaging for perishable fruits preservation. Int. J. Biol. Macromol..

[140] Yang J., Dahlström C., Edlund H., Lindman B., Norgren M. (2019). pH-responsive cellulose–chitosan nanocomposite films with slow release of chitosan. Cellulose.

[141] Du C., Li S., Fan Y., Lu Y., Sheng J., Song Y. (2023). Preparation of gelatin-chitosan bilayer film loaded citral nanoemulsion as pH and enzyme stimuli-responsive antibacterial material for food packaging. Int. J. Biol. Macromol..

[142] Ma Y., Wang S., Zhang Z., Cao X., Zhang B., Wu D., Chen K., Wang W.-J., Liu P. (2022). Grafting Hollow Covalent Organic Framework Nanoparticles with Thermal-Responsive Polymers for the Controlled Release of Preservatives. ACS Appl. Mater. Interfaces.

[143] Mao S., Li F., Zhou X., Lu C., Zhang T. (2023). Characterization and sustained release study of starch-based films loaded with carvacrol: A promising UV-shielding and bioactive nanocomposite film. LWT—Food Sci. Technol..

[144] Wang X., Zhu J., Tang T., Yang L., Chen X., Meng S., Zheng R., Wu H. (2024). Carboxymethyl chitosan coating infused with linalool-loaded molten globular β-Lactoglobulin nanoparticles for extended preservation of fresh-cut apples. Food Chem..

[145] Xiao Y., Ahmad T., Belwal T., Aadil R.M., Siddique M., Pang L., Xu Y. (2023). A review on protein based nanocarriers for polyphenols: Interaction and stabilization mechanisms. Food Innov. Adv..

[146] Rehman Sheikh A., Wu-Chen R.A., Matloob A., Mahmood M.H., Javed M. (2024). Nanoencapsulation of volatile plant essential oils: A paradigm shift in food industry practices. Food Innov. Adv..

[147] Bi J., Fang H., Zhang J., Lu L., Gu X., Zheng Y. (2023). A review on the application, phytochemistry and pharmacology of Polygonatum odoratum, an edible medicinal plant. J. Future Foods.

[148] Chen Y., Yu W., Zhang S., Niu Y., Zhang Y., Liu T. (2023). Pressure shear assisted extraction of polysaccharide from Auricularia auricula and its biological activities. J. Future Foods.

[149] Lang Y., Gao N., Zang Z., Meng X., Lin Y., Yang S., Yang Y., Jin Z., Li B. (2024). Classification and antioxidant assays of polyphenols: A review. J. Future Foods.

[150] Nayak A., Mukherjee A., Kumar S., Dutta D. (2024). Exploring the potential of jujube seed powder in polysaccharide based functional film: Characterization, properties and application in fruit preservation. Int. J. Biol. Macromol..

[151] Huang G., Huang L., Geng C., Lan T., Huang X., Xu S., Shen Y., Bian H. (2022). Green and multifunctional chitosan-based conformal coating as a controlled release platform for fruit preservation. Int. J. Biol. Macromol..

[152] Li X., He J., Zhang W., Khan M.R., Ahmad N., Tian W. (2024). Pectin film fortified with zein nanoparticles and Fe3+-Encapsulated propolis extract for enhanced fruit preservation. Food Hydrocoll..

[153] Zhang L., He W., Hu P., Wang W., Luo L., Li B., Pan B., Zhu W., Wang Y., Wang J. (2024). Self-assembly technology engineering multi-functional slow-release packaging system with self-starting program for prolonged preservation of perishable products. Food Hydrocoll..

[154] Zou J., Zhong H., Jiang C., Zhu G., Lin X., Huang Y. (2024). Ginkgo biloba leaf polysaccharide-stabled selenium nanozyme as an efficient glutathione peroxidase mimic for the preservation of bananas and cherry tomatoes. Food Chem..

[155] Qi C., Dianpeng H., Xiaoyu Q., Wen Z., Yuan P., Shuang L., Kang Q., Shuyue R., Yu W., Huanying Z. (2025). Self-enhanced multifunctional composite membranes based on metal-organic frameworks embedded with thymol nanoparticles for post-harvest preservation of fruits. Food Hydrocoll..

[156] Jiang T.-S., Li X.-Y., Zhu C.-H., Yu T.-R., Zhao H.-Q. (2024). Bismuth selenide nanosheet layer materials with peroxidase activity for antimicrobial applications. Adv. Agrochem..

[157] Zhang S., Fu Q., Li H., Li Y., Wu P., Ai S. (2024). Polydopamine-coated lignin nanoparticles in polysaccharide-based films: A plasticizer, mechanical property enhancer, anti-ultraviolet agent and bioactive agent. Food Hydrocoll..

[158] Silva N.H.C.S., Vilela C., Almeida A., Marrucho I.M., Freire C.S.R. (2018). Pullulan-based nanocomposite films for functional food packaging: Exploiting lysozyme nanofibers as antibacterial and antioxidant reinforcing additives. Food Hydrocoll..

[159] Ding X., Lin H., Zhou J., Lin Z., Huang Y., Chen G., Zhang Y., Lv J., Chen J., Liu G. (2024). Silver Nanocomposites with Enhanced Shelf-Life for Fruit and Vegetable Preservation: Mechanisms, Advances, and Prospects. Nanomaterials.

[160] Dong H., Xu Y., Zhang Q., Li H., Chen L. (2024). Activity and safety evaluation of natural preservatives. Food Res. Int..

[161] Costa R.R., Neto A.I., Calgeris I., Correia C.R., Pinho A.C.M., Fonseca J., Öner E.T., Mano J.F. (2013). Adhesive nanostructured multilayer films using a bacterial exopolysaccharide for biomedical applications. J. Mater. Chem. B.

[162] Yu Y., Zhou J., Chen Q., Xie F., Zhang D., He Z., Cheng S., Cai J. (2024). Self-reinforced multifunctional starch nanocomposite film for litchi fruit postharvest preservation. Chem. Eng. J..

[163] Qin C.C., Abdalkarim S.Y.H., Yang M.C., Dong Y.J., Yu H.-Y., Ge D. (2023). All-naturally structured tough, ultrathin, and washable dual-use composite for fruits preservation with high biosafety evaluation. Int. J. Biol. Macromol..

[164] Efthymiou M.-N., Tsouko E., Papagiannopoulos A., Athanasoulia I.-G., Georgiadou M., Pispas S., Briassoulis D., Tsironi T., Koutinas A. (2022). Development of biodegradable films using sunflower protein isolates and bacterial nanocellulose as innovative food packaging materials for fresh fruit preservation. Sci. Rep..

[165] Mondéjar-López M., Castillo R., Jiménez A.J.L., Gómez-Gómez L., Ahrazem O., Niza E. (2023). Polysaccharide film containing cinnamaldehyde-chitosan nanoparticles, a new eco-packaging material effective in meat preservation. Food Chem..

[166] Li X., Li F., Zhang X., Tang W., Huang M., Huang Q., Tu Z. (2024). Interaction mechanisms of edible film ingredients and their effects on food quality. Curr. Res. Food Sci..

[167] Andreica B.-I., Cheng X., Marin L. (2020). Quaternary ammonium salts of chitosan. A Critical Overview on the Synthesis and Properties Generated by Quaternization. Eur. Polym. J..

[168] Wu K.C., Freedman B.R., Kwon P.S., Torre M., Kent D.O., Bi W.L., Mooney D.J. (2024). A tough bioadhesive hydrogel supports sutureless sealing of the dural membrane in porcine and ex vivo human tissue. Sci. Transl. Med..

[169] Bhattacharjee B., Tabbasum K., Mukherjee R., Garg P., Haldar J. (2024). Functionalized chitosan based antibacterial hydrogel sealant for simultaneous infection eradication and tissue closure in ocular injuries. Int. J. Biol. Macromol..

[170] Eshkol-Yogev I., Gilboa E., Giladi S., Zilberman M. (2021). Formulation—Properties effects of novel dual composite hydrogels for use as medical sealants. Eur. Polym. J..

[171] Dilara D., Mustafa T., Funda K. (2022). Antifungal Activities of Different Essential Oils and Their Electrospun Nanofibers against Aspergillus and Penicillium Species Isolated from Bread. ACS Omega.

[172] Wang S., Li M., He B., Yong Y., Zhu J. (2023). Composite films of sodium alginate and konjac glucomannan incorporated with tea polyphenols for food preservation. Int. J. Biol. Macromol..

[173] Su W., Chang Z., E Y.Y., Feng Y., Yao X., Wang M., Ju Y., Wang K., Jiang J., Li P. (2024). Electrospinning and electrospun polysaccharide-based nanofiber membranes: A review. Int. J. Biol. Macromol..

[174] Wu H., Wang X., Li S., Zhang Q., Chen M., Yuan X., Zhou M., Zhang Z., Chen A. (2024). Incorporation of cellulose nanocrystals to improve the physicochemical and bioactive properties of pectin-konjac glucomannan composite films containing clove essential oil. Int. J. Biol. Macromol..

[175] Zhou W., He Y., Liu F., Liao L., Huang X., Li R., Zou Y., Zhou L., Zou L., Liu Y. (2020). Carboxymethyl chitosan-pullulan edible films enriched with galangal essential oil: Characterization and application in mango preservation. Carbohydr. Polym..

[176] Ezati P., Rhim J.-W. (2020). pH-responsive chitosan-based film incorporated with alizarin for intelligent packaging applications. Food Hydrocoll..

[177] Yin S., Duan M., Fellner M., Wang Z., Lv C., Zang J., Zhao G., Zhang T. (2024). pH/glucose dual-responsive protein-based hydrogels with enhanced adhesive and antibacterial properties for diabetic wound healing. Food Innov. Adv..

[178] Huang H.-L., Tsai I.L., Lin C., Hang Y.-H., Ho Y.-C., Tsai M.-L., Mi F.-L. (2022). Intelligent films of marine polysaccharides and purple cauliflower extract for food packaging and spoilage monitoring. Carbohydr. Polym..

[179] Puttipan R., Khankaew S. (2023). Novel bio-based, time-temperature dependent colorimetric ink and film containing colorants from the red pitaya (*Hylocereus costaricensis*). Prog. Org. Coat..

[180] Yuan L., Gao M., Xiang H., Zhou Z., Yu D., Yan R. (2023). A Biomass-Based Colorimetric Sulfur Dioxide Gas Sensor for Smart Packaging. ACS Nano.

[181] Wu Y., Li B.-H., Chen M.-M., Bing L. (2024). An aerogel-based intelligent active packaging with the dual functions of spoilage detection and freshness preservation. Food Hydrocoll..

[182] Mohammadian E., Alizadeh-Sani M., Jafari S.M. (2020). Smart monitoring of gas/temperature changes within food packaging based on natural colorants. Compr. Rev. Food Sci. Food Saf..

[183] Rai P., Mehrotra S., Sharma S.K. (2024). Potential of sensing interventions in the life cycle assessment of fruits and fruit juices. Trends Food Sci. Technol..

[184] Zheng Q., Bai X., Chen T., Li F., Zhu P., Li M., Tang Y. (2024). Carboxymethyl hemicellulose/sorbitol/gallic acid green composite films for fresh fruit preservation. Ind. Crops Prod..

[185] Guo X., Li L., Qi Y., Su J., Ou X., Lv M., Jin Y., Han X., Zhang Y., Wu H. (2024). Green-synthesized antibacterial and unidirectional water-permeable polylactic acid/ZnO composite film for enhanced preservation of perishable fruits. Mater. Today Chem..

[186] Xi W., Liu P., Ling J., Xian D., Wu L., Yuan Y., Zhang J., Xie F. (2023). Pre-gelatinized high-amylose starch enables easy preparation of flexible and antimicrobial composite films for fresh fruit preservation. Int. J. Biol. Macromol..

[187] Ali M.H., Dutta S.K., Sultana M.S., Habib A., Dhar P.K. (2024). Green synthesized CeO_2_ nanoparticles-based chitosan/PVA composite films: Enhanced antimicrobial activities and mechanical properties for edible berry tomato preservation. Int. J. Biol. Macromol..

[188] Heras M., Huang C.-C., Chang C.-W., Lu K.-H. (2024). Trends in chitosan-based films and coatings: A systematic review of the incorporated biopreservatives, biological properties, and nanotechnology applications in meat preservation. Food Packag. Shelf Life.

